# Metabolite Profiling Analysis and the Correlation with Biological Activity of Betalain-Rich *Portulaca grandiflora* Hook. Extracts

**DOI:** 10.3390/antiox11091654

**Published:** 2022-08-25

**Authors:** Aneta Spórna-Kucab, Anna Tekieli, Agnieszka Grzegorczyk, Łukasz Świątek, Barbara Rajtar, Krystyna Skalicka-Woźniak, Karolina Starzak, Boris Nemzer, Zbigniew Pietrzkowski, Sławomir Wybraniec

**Affiliations:** 1 Department of Chemical Technology and Environmental Analysis, Faculty of Chemical Engineering and Technology, Cracow University of Technology, Warszawska 24, 31-155 Krakow, Poland; 2 Chair and Department of Pharmaceutical Microbiology, Medical University of Lublin, 1 Chodźki Str, 20-093 Lublin, Poland; 3 Department of Virology with SARS Laboratory, Medical University of Lublin, 1 Chodźki Str, 20-093 Lublin, Poland; 4 Department of Natural Products Chemistry, Medical University of Lublin, 1 Chodźki Str, 20-093 Lublin, Poland; 5 Research and Analytical Center, VDF FutureCeuticals, Inc., 2692 N. State Rt. 1-17, Momence, IL 60954, USA; 6 Food Science & Human Nutrition, University of Illinois at Urbana-Champaign, 260 Bevier Hall, 905 S. Goodwin Ave., Urbana, IL 61801, USA; 7 Discovery Research Lab, VDF FutureCeuticals, Inc., 23 Peters Canyon Rd., Irvine, CA 92606, USA

**Keywords:** antimicrobial, antioxidant, antiviral, betalains, cytotoxicity, *Portulaca grandiflora* Hook.

## Abstract

The aim of the study was to evaluate the possible correlation between the bioactivity and the phytochemical profile of four betalain-rich extracts from *Portulaca grandiflora* Hook. The HPLC-DAD-ESI-MS analysis indicated the presence of 19 betaxanthins and two betacyanins. The highest concentrations of betaxanthins (982 mg/100 g DE) and betacyanins (650 mg/100 g DE) were noticed in orange and purple flowers extracts, respectively. The HPLC-DAD-ESI-HRMS/MS analyses revealed the presence of a total of 71 compounds. Fifteen new betaxanthins and fifty other metabolites were identified for the first time. The antioxidant activity of the studied flower extracts increased in the sequence of yellow < orange < purple < red (0.066–0.176 mM TE/g DE). Betalains showed less effect on the antioxidant activity of extracts than other metabolites did. Extracts from yellow and orange flowers were more active against Gram-positive bacteria (MIC = 4–16 mg/L), whereas extracts from red and purple flowers were slightly more active against Gram-negative bacteria (MIC = 16–32 mg/L). All the extracts showed the same activity against yeasts (MIC = 32 mg/L). Betaxanthins were active against Gram-positive bacteria, whereas betacyanins were active against Gram-negative bacteria. Remaining metabolites also exhibited antimicrobial activities. The cytotoxicity assessment showed that the *P. grandiflora* extracts were non-toxic to normal VERO cells. No significant antiviral activity towards Human Herpesvirus type 1 was observed (62 µg/mL). Among the tested varieties, the purple one showed anticancer selectivity towards colon carcinoma cells (RKO).

## 1. Introduction

*Portulaca grandiflora* Hook. (Portulacaceae) is known as one of the plants with beneficial properties for health [1,2,3,4]. *P. grandiflora* is used for the relief of sore throats and skin rashes as well as for detoxification [1,3,5]. It is a putative immunostimulant [1] and induces a nonspecific activation of the immune response including cytotoxic, antimicrobial and anticancer activity [6,7]. *P. grandiflora* has been reported for its efficacy on a hepatitis B virus surface antigen. Moreover, the antimutagenic effect of this plant on the mutation induced by human carcinogens, aflatoxin B1 and cyclophosphamide was reported in mice [3,8]. It is worth emphasizing that *P. grandiflora* aqueous extracts can be used safely; clinical trials indicated the acceptable dose was 500 mg/day [3].

*P. grandiflora* flowers produce and accumulate betalains, which are divided into yellow-orange betaxanthins and red-violet betacyanins [9,10,11,12,13,14,15]. Numerous studies have demonstrated the health benefits of betalains arising from their high antioxidant activity and this is because these compounds have been found to be active against stress-related disorders in humans due to their potential to inhibit lipid oxidation and peroxidation [16,17,18,19,20]. Betacyanins have been found to protect LDLs (low-density lipoproteins) from oxidation. High levels of oxidized LDLs in the blood are dangerous to health as they can lead to the development of cardiovascular disease, a heart attack and a stroke. Moreover, the protective effect of betalains against DNA-damage was noticed [21].

Considerable interest in these compounds is a result of their potential anticancer, antimicrobial, antiviral as well as anti-inflammatory and neuroprotective actions [16,21,22,23,24]. Betalains are considered as cytostatics which are used effectively in cancer chemotherapy. The conducted studies confirm their toxic effect on colorectal cancer cells, human lymphoma, human melanoma (B16F10), leukemia (K562), human liver cancer (HepG2), human liver hepatoma (Huh7) and breast cancer (MCF-7) [15,22]. Moreover, it has been noticed that betalains have a potential use as antiviral agents in the therapy against dengue virus (DENV) [21]. The antibacterial and antifungal efficiency of betacyanins was confirmed for pure compounds that were isolated from purple *Gomphrena globosa* L. [25]. Clinical studies of the effect of betalain-rich extracts confirm their pro-health activities [23].

Plants are very complex matrices including many bioactive compounds. Except betalains, other plant-derived metabolites also indicate numerous health-related effects, including antimicrobial, antioxidant, anticancer, anti-inflammatory, antidepressant, etc. Food that is rich in bioactive compounds is considered as functional food which provides health benefits beyond the provision of essential nutrients [26]. Herein, a strategy for the rapid detection and identification of the metabolites, including the betalains of *P. grandiflora*, by employing a HPLC-DAD-ESI-HRMS/MS analysis was established. To the best of our knowledge, this study presents the first metabolite profile of *P. grandiflora*. The presence and concentration of chemical substances have an influence on the biological activity of the plants therefore, this report is aimed at evaluating the correlation between the phytochemical profile and biological properties of the four extracts obtained from fresh yellow, orange, red and purple flowers of *P. grandiflora*. Our study will be helpful to clarify which compounds are responsible for the biological activity of the *P. grandiflora*.

## 2. Materials and Methods

### 2.1. Reference Compounds and Reagents

Reference betalains were derived from extracts of ripe cactus fruits of *Hylocereus polyrhizus* [27] as well as red roots of *Beta vulgaris* L. [28].

Acetone (HPLC-grade), potassium persulfate, sodium acetate trihydrate and ascorbic acid (pure p. a.) as well formic acid (purity ≥ 98%) and hydrochloric acid (pure p. a.) were obtained from Avantor Performance Materials Poland S.A. (Gliwice, Poland).

Methanol (LC-MS-grade) and ABTS (2,2’-Azino-bis(3-ethylbenzthiazoline-6-sulfonic acid), trolox (6-Hydroxy-2,5,7,8-tetramethylchroman-2-carboxylic acid), DPPH (2,2-diphenyl-1-picrylhydrazyl), TPTZ (2,4,6-tris(2-pyridyl)-*s*-triazine) and iron (III) chloride hexahydrate were obtained from Sigma-Aldrich (Saint Louis, MO, USA).

The VERO cells (ATCC, Cat. No. CCL-81) were cultured using Dulbecco Modified Eagle Medium (DMEM, Corning, Tewksbury, MA, USA), whereas HeLa (ECACC, Cat. No. 93021013) and RKO (ATCC, Cat. No. CRL-2577) were cultured using Modified Eagle Medium (MEM). All media were supplemented with antibiotics (Penicillin-Streptomycin Solution, Corning, New York, USA) and fetal bovine serum (FBS, Capricorn, Ebsdorfergrund, Germany). The PBS (phosphate buffered saline) and trypsin were obtained from Corning, whereas MTT (3-(4,5-dimethylthiazol-2-yl)-2,5-diphenyltetrazolium bromide), acyclovir and DMSO (dimethyl sulfoxide) were obtained from Sigma (Sigma-Aldrich, Saint Louis, MO, USA). 

Reference microorganisms from ATCC (American Type Culture Collection, LGC Standards, Teddington, UK): *Staphylococcus aureus* ATCC 25923, *Staphylococcus aureus* ATCC 6538, *Staphylococcus aureus* ATCC 29213, *Staphylococcus aureus* ATCC BAA-1707, *Staphylococcus epidermidis* ATCC 12228, *Micrococcus luteus* ATCC 10240, *Bacillus subtilis* ATCC 6633, *Bacillus cereus* ATCC 10876, *Escherichia coli* ATCC 25922, *Salmonella typhimurium* ATCC 14028, *Pseudomonas aeruginosa* ATCC 27852, *Candida albicans* ATCC 10231, *Candida glabrata* ATCC 90030 and *Candida krusei* ATCC 14243 were used in the study. Vancomycin, ciprofloxacin and fluconazole were obtained from Sigma (Sigma-Aldrich, Saint Louis, MO, USA). 

### 2.2. Plant Material and Sample Preparation

Seeds of *P. grandiflora* were bought from Polan Company (Cracow, Poland) in April 2019. The plants have been grown since May 2019. The flowers with yellow, orange, red and purple flowers were harvested separately in the period from July to September 2019 and directly frozen.

*P. grandiflora* fresh flowers (100 g) were extracted separately by maceration using a mixture of water/acetone/formic acid (59/40/1, *v*/*v*/*v*) for 30 min at room temperature. The obtained crude extracts were centrifuged at 4000 rpm for 5 min and then, the extracts were partially evaporated at 25 °C under reduced pressure (Büchi, Flawil, Switzerland) and freeze-dried (Christ, Benningen, Germany). The dried extracts were weighed and used in further research on their antioxidant, antimicrobial and antiviral activities as well as a cytotoxicity evaluation.

### 2.3. Total Content of Betalain

The total betalain (betacyanins and betaxanthins) concentration was measured spectrophotometrically using the Nilsson method [29] using a microplate reader (Infinite M200, Tecan, Grödig, Austria). Betaxanthin and betacyanin contents were measured to be 474 and 538 nm, respectively. Total betaxanthins and betacyanins contents were expressed as mg of compound in 100 g of dry extract (mg/100 g DE). The molar extinction coefficient (ε) value for betaxanthins is 4.80 × 10^4^ cm^−1^ mol^−1^ L, and for betacyanins this is 6.16 × 10^4^ cm^−1^ mol^−1^ L [30]. Measurements of the absorption values for yellow, orange, red and purple extracts of *P. grandiflora* were performed in triplicate. 

### 2.4. Betalain Profile by HPLC-DAD-ESI-MS Analysis 

The qualitative and quantitative determination of single betalains (betacyanins and betaxanthins) was carried out using an HPLC-DAD-ESI-MS system (LCMS-8030, Shimadzu, Kyoto, Japan) equipped with a quadrupole mass spectrometer and diode-array (DAD) detectors and controlled by a LabSolutions system. The electrospray ion (ESI) source was operating in the positive ionization mode due to the ionization of betalains in this mode. 

For HPLC experiments, the column used was a Kinetex C_18_ (100 mm × 4.6 mm I.D. × 5.0 μm) that was protected by a guard column (Phenomenex, Torrance, CA, USA) at 40 °C with a mobile phase composed of 2% formic acid in water (solvent A) and methanol (solvent B) in the following gradients: 0–12 min, 1–11% B; 12–24 min, 11–60% B; 24–24.1 min, 60–90% B. The flow rate was 0.5 mL/min, while the sample injection was 15 μL. The UV-Vis spectra were recorded in the range 200–800 nm.

MS operating parameters were applied: mass-to-charge (*m*/*z*) range 100–2000; drying gas (nitrogen); curved desolvation line (CDL) and heat block temperature 230 °C; nebulizing gas flow rate 1.5 L/min; electrospray voltage of 4.5 kV; capillary temperature 250 °C. 

To confirm the identified betalains and the retention time, mass and UV-Vis spectra were compared with reference standards from ripe cactus fruits of *Hylocereus polyrhizus* as well as red roots of *Beta vulgaris* L. The content of single betalains was estimated from the peak areas using MS chromatograms of studied *P. grandiflora* extracts. Reference standards for individual betalains are not available due to their lower stability beyond the plant matrix.

### 2.5. Phytochemical Profile by HPLC-DAD-ESI-HRMS/MS Analysis

The purified samples were analysed qualitatively using an HPLC/ESI-QTOF-MS system in positive and negative ion modes with the use of a 6530B Accurate-mass-QTOF-MS (Agilent Technologies, Inc., Santa Clara, CA, USA) mass spectrometer with an ESI-Jet Stream ion source. The Agilent 1260 chromatograph was equipped with a DAD detector, autosampler, binary gradient pump, and column oven. The following gradient of solvents were used for the mobile phases: water with 0.1% formic acid (solvent A) and acetonitrile with 0.1% formic acid (solvent B). The following gradient procedure was adopted: 0–45 min, 0–60% of B; 45–46 min, 60–95% B; 46–55 min 95% (B), and the post time was 10 min. Total time of analysis was 65 min, with a stable flow rate at 0.200 mL/min. Injection volume for extracts was 10 μL. ESI-QToF-MS analysis was performed according to the following parameters of the ion source: dual spray jet stream ESI; positive and negative ion mode; gas (N_2_) flow rate: 12 L/min; nebulizer pressure: 35 psi; vaporizer temp.: 300 °C; *m*/*z* range 100–1000 mass units with acquisition Mode Auto MS/MS; collision induced dissociation (CID): 10 and 30 eV with MS scan rate 1 spectrum per s, 2 spectra per cycle; skimmer: 65 V; fragmentor: 140 V and octopole RF Peak: 750 V.

### 2.6. Antioxidant Activity

#### 2.6.1. ABTS Radical Scavenging Assay

The antioxidant capacity in vitro of *P. grandiflora* flowers was measured spectrophotometrically using ABTS^•+^ radicals as well as reference compounds, trolox and ascorbic acid. ABTS^•+^ is a stable, water-soluble radical cation obtained during the reaction of ABTS with sodium persulfate (K_2_S_2_O_8_) in the dark for 8–16 h. Prepared radical solution should be protected from direct sunlight and kept in the refrigerator for longer storage. 

Increasing volumes of aqueous extracts (1 mg/mL) of *P. grandiflora* were applied to the wells of transparent 96-well plates so that their final volume was set to decrease the radicals’ absorbance in the range of 10–90% of its initial intensity. The final concentration of extracts ranged from 0 to 0.7 mg/mL in 200 μL of the total volume of each sample. Trolox (0.025 mg/mL) and ascorbic acid (0.020 mg/mL) were prepared in the same way. 

Prior to the measurement, all microplate wells were supplemented with 40 μL of 1 mM aqueous solution of ABTS^•+^ radicals. To ensure thorough mixing of reagents in all microplate wells, each plate was shaken on an internal shaker on the reader for 10 s. The spectrophotometric measurements were performed at λ 734 nm at 20 °C after 30 min of reaction and while they were kept in the dark, on a microplate reader (Infinite M200, Tecan, Grödig, Austria). The results obtained were the average of five exposures of each sample with a beam of light. All experiments were replicated three times. The results were reported as mM trolox equivalent per gram of dry extract (mM TE/g DE), which specifies how many times the given extract potential is higher or lower than the standard.

#### 2.6.2. DPPH Radical Scavenging Assay

The antioxidant activity in vitro of *P. grandiflora* flowers was also assessed with use of the spectrophotometric method DPPH based on a reduction of DPPH radicals at room temperature. DPPH is a relatively stable radical that has a violet colour in methanol solution with an absorption maximum of 515 nm. During the reaction with a hydrogen donor, they turn into a reduced form, and the solution becomes yellow-orange. 

To a transparent, 96-well plate, increasing concentrations of aqueous extracts (1 mg/mL) of *P. grandiflora* were added so that their final volume was set to decrease the radicals’ absorbance in the range of 10–90% of its initial intensity. Then, 20 µL of water and 40 µL of 1.0 mM DPPH^•^ radicals methanolic solution were applied. The final concentration of extracts ranged from 0 to 0.6 mg/mL in 200 μL of the total volume of each sample. Samples of the reference compounds, which were trolox (0.025 mg/mL) and ascorbic acid (0.020 mg/mL), were prepared in the same way. The plate was shaken for 10 s on the reader’s shaker to obtain homogeneous solutions. The spectrophotometric measurements were performed at λ 515 nm at 20 °C after 30 min of reaction and while they were kept in the dark, on a microplate reader (Infinite M200, Tecan, Grödig, Austria). The results obtained were the average of five exposures of each sample with a beam of light. All experiments were replicated three times. The results were reported as mM trolox equivalent per gram of dry extract (mM TE/g DE), which specifies how many times the given extract potential is higher or lower than the standard.

#### 2.6.3. FRAP Ferric Reducing Antioxidant Power Assay

The antioxidant activity in vitro of *P. grandiflora* flowers was also measured with use of the spectrophotometric method FRAP according to the methodology proposed previously [31], with minor modifications. This assay is based on the reduction, at low pH, of a colourless ferric complex (Fe(TPTZ)_2_)^3+^ to a dark blue-coloured ferrous complex (Fe(TPTZ)_2_)^2+^ containing the 2,4,6-tris(2-pyridyl)-*s*-triazine ligand (TPTZ) by the action of electron-donating antioxidants [32,33].

FRAP reagent was freshly prepared by mixing 300 mM of buffer acetate pH 3.6 with 20 mM of FeCl_3_·6H_2_O and 10 mM of TPTZ dissolved in 40 mM of hydrochloric in ratios of 10:1:1 (*v*/*v*/*v*), respectively. In 96-well plates, 133 µL of a freshly prepared FRAP reagent was added to each sample (40 µL for yellow, orange, red extracts of *P. grandiflora* and 20 µL for purple) separately (1.5 g/mL) in the total volume 200 µL. Samples of the reference compounds, which were trolox (0.050 mg/mL) and ascorbic acid (0.020 mg/mL), were prepared in the same way. The plates were shaken for 10 s on the reader shaker to obtain homogeneous solutions. The spectrophotometric measurements were performed at λ 593 nm at 20 °C after 10 min of reaction and while they were kept in the dark, on a microplate reader (Infinite M200, Tecan, Grödig, Austria). The results obtained were the average of five exposures of each sample with a beam of light. All experiments were replicated three times. The results were reported as mM trolox equivalent per gram of dry extract (mM TE/g DE), which specifies how many times the given extract potential is higher or lower than the standard.

### 2.7. Antimicrobial Activity

The assay of antibacterial and antifungal activities of the extracts from the yellow, orange, red and purple flowers obtained from *P. grandiflora* was performed using the broth microdilution method according to EUCAST (the European Committee on Antimicrobial Susceptibility Testing) recommendations and the modified method by Malm and Grzegorczyk [34,35]. The following reference strains from ATCC (American Type Culture Collection) were used in the study: *Staphylococcus aureus* ATCC 25923, *Staphylococcus aureus* ATCC 6538, *Staphylococcus aureus* ATCC 29213, *Staphylococcus aureus* ATCC BAA-1707, *Staphylococcus epidermidis* ATCC 12228, *Micrococcus luteus* ATCC 10240, *Bacillus subtilis* ATCC 6633, *Bacillus cereus* ATCC 10876 (representative of Gram-positive bacteria), *Escherichia coli* ATCC 25922, *Salmonella typhimurium* ATCC 14028, *Pseudomonas aeruginosa* ATCC 27852 (representative of Gram-negative bacteria), *Candida albicans* ATCC 10231, *Candida glabrata* ATCC 90030 and *Candida krusei* ATCC 14243 (representatives of yeast fungi). All the used microbial strains were first subcultured in Mueller-Hinton Agar (MHA for bacteria) or Mueller-Hinton Agar with 2% glucose (MHA + 2% glucose for fungi) and incubated at 35 ± 1 °C for 18 ± 2 h. Microbial colonies were collected and suspended in sterile physiological saline to obtain an inoculum of 0.5 McFarland standard, corresponding to 1.5 × 10^8^ CFU (colony forming units)/mL for bacteria and 5 × 10^6^ CFU/mL for fungi. The extracts were dissolved in sterile distilled water to obtain the final concentration 100 mg/mL. The 2-fold dilutions of extracts in Mueller-Hinton Broth (MHB for bacteria) or in Mueller-Hinton Broth with 2% glucose (MHB + 2% glucose for fungi) were prepared in 96-well polystyrene plates to obtain final concentrations ranging from 32 to 0.125 mg/mL. Next, 2 µL of a particular bacterial or fungal inoculum was added to each well containing 200 µL of the serial dilution of extracts in the appropriate culture medium. After incubation at 35 ± 1 °C for 18 ± 2 h, the MIC (minimum inhibitory concentration) was assessed spectrophotometrically with the lowest concentration of extracts showing complete bacterial or fungal growth inhibition. The MBCs (minimum bactericidal concentrations) or MFCs (minimum fungicidal concentrations) were determined by removing 5 μL of the bacterial or fungal culture used for the MIC determinations from each well and spotting this onto an appropriate agar medium. The plates were incubated at 35 ± 1 °C for 18 ± 2 h. The lowest extract concentrations with no visible bacterial or fungal growth were assessed as MBC or MFC, respectively. Vancomycin (range of 0.06–16 μg/mL), ciprofloxacin (range of 0.015–16 μg/mL) and fluconazole (range of 0.06–16 μg/mL) were included as the reference antimicrobial substances active against Gram-positive bacteria, Gram-negative bacteria and yeasts. The experiments were performed in triplicate. Of the three MIC, MBC and MFC values, the most common representative value was presented.

### 2.8. Cytotoxicity Evaluation

The cytotoxicity was tested towards the VERO, HeLa and RKO cell lines using an MTT based assay. Incubation was carried out in 5% CO_2_ atmosphere at 37 °C (CO_2_ incubator, Panasonic Healthcare Co., Tokyo, Japan). The cells were passaged into 96-well plates and incubated overnight to produce a semi-confluent monolayer. Afterwards, cells were treated with a series of dilutions (4–8000 µg/mL for VERO or 4–4000 µg/mL for cancer cells) of tested extracts in cell media for 24 h or 72 h. Subsequently, cell media was removed from the plates, the wells were washed with PBS, and 10% of MTT solution (5 mg/mL) in fresh cell media was added and incubated for the next 4 h to allow cellular dehydrogenases to reduce yellow 3-(4,5-dimethylthiazol-2-yl)-2,5-diphenyltetrazolium bromide to violet formazan. Finally, the SDS/DMF/PBS (14% SDS, 36% DMF, 50% PBS) solvent was added, 100 µL per well, to dissolve the precipitated formasane and after overnight incubation 37 °C, the absorbance was measured at λ 540 and 620 nm using Synergy H1 Multi-Mode Microplate Reader (BioTek Instruments, Inc. Winooski, VT, USA) equipped with Gen5 software (ver. 3.09.07; BioTek Instruments, Inc.). The obtained data were further analysed using GraphPad Prism (v7.04, GraphPad, San Diego, CA, USA).

### 2.9. Antiviral Activity

#### 2.9.1. The Effect of *Portulaca grandiflora* Hook. on HHV-1 Induced Cytopathic Effect

The infectious titer of HHV-1 (ATCC, No. VR-260, Human herpesvirus 1, Herpes simplex virus 1, HSV-1) that was used for antiviral studies was 5.5 ± 0.25 logCCID_50_/mL (CCID_50_—50% cell culture infectious dose). The VERO cells were passaged into 48-well plates and incubated overnight. Afterwards, the cells were treated with HHV-1 at a 100-fold CCID_50_ dose and incubated for 1 h to allow for the virus’ adsorption, washed with PBS, and then tested extracts in the concentration of 62 µg/mL were added, and the incubation continued until the cytopathic effect (CPE) was observed in non-treated virus infected cells (virus control). The plates were examined using an inverted microscope (CKX41, Olympus Corporation, Tokyo, Japan) equipped with a camera (Moticam 3+, Motic, Hong Kong), and the influence of the tested extracts on CPE formation in comparison with the CPE that was observed for the virus control was documented (Motic Images Plus 2.0, Motic, Kowloon, Hong Kong). Finally, the 48-well plates were thrice frozen (−72 °C) and thawed, the samples were collected and stored at −72 °C until they were used for the end-point virus titration assay and viral DNA isolation. 

#### 2.9.2. End-Point Dilution Test for HHV-1 Titration

To perform the end-point virus titration test, the VERO cells growing in 96-well plates were treated with tenfold dilutions of samples collected during the evaluation of antiviral activity and incubated with cells for 72 h. Afterwards, the media were removed from the plates and the HHV-1 infectious titer in samples was measured using MTT method, as described, for cytotoxicity. The measure of antiviral activity was calculated as the difference of HHV-1 infectious titer (logCCID_50_) in the samples collected from tested extracts (TE) in comparison with the virus control (VC) from the same experiment, which was expressed as Δlog (Δlog = logCCID_50_VC − logCCID_50_TE). The end-point virus titration was done for every antiviral assay performed and the results were calculated as a means of viral titer reduction. In the studies of antiviral activity, a significant effect can be reported for samples decreasing the virus infectious titer by at least three log in comparison to the virus control.

#### 2.9.3. The DNA Isolation and Real-Time PCR Analysis for HHV-1 Viral Load

The DNA was isolated using QIAamp DNA Mini Kit (Cat#51304, QIAGEN GmbH, Hilden, Germany) according to the manufacturer’s protocol. The Real-Time PCR (qPCR) amplification was performed using a Rotor-Gene Q (QIAGEN) thermal cycler using TB Green Advantage qPCR Premixes (Takara Bio, Mountain View, CA, USA) and primers (UL54F–5′ CGCCAAGAAAATTTCATCGAG 3′, UL54R–5′ ACATCTTGCACCACGCCAG 3′) for the UL54 coding region (encoding ICP27—A regulatory protein required for HHV-1 infection). These were the amplification parameters: initial denaturation (95 °C, 20 s); cycling (45 repeats: denaturation (95 °C, 5 s); annealing/extension (60 °C, 30 s); fluorescence acquisition (Green); melting curve analysis (60–95 °C). The viral load was measured with reference to the calibration curve. The calibration curve comprised of tenfold dilutions of stock virus DNA isolate quantitatively analysed using IVD certified GeneProof Herpes Simplex Virus (HSV-1/2) PCR Kit (Cat#HSV/ISEX/025, GeneProof a.s., Brno, Czech Republic), following the manufacturer’s procedure.

### 2.10. Statistical Analysis

One-way analysis of variance (ANOVA) was used for the statistical analysis of the means of four extracts of yellow, orange, red and purple varieties of *P. grandiflora*, with the help of Statistica, version 7.1 (StatSoft, TIBCO Software Inc. Palo Alto, CA, USA). Results were subjected to ANOVA, and differences between means were located using Fisher’s test. Significance was determined at the α level, 0.05 to find out how many and which cultivars have different contents. Data were reported as the mean ± standard deviation (SD) of three measurements.

## 3. Results and Discussion

### 3.1. Phytochemical Analyses

**Figure 1:**
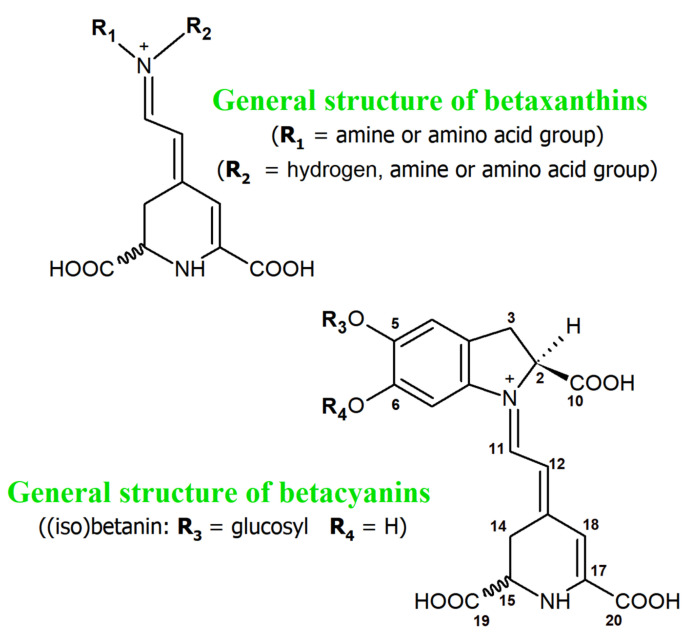
General chemical structures of betacyanins and betaxanthins.

**Figure 2:**
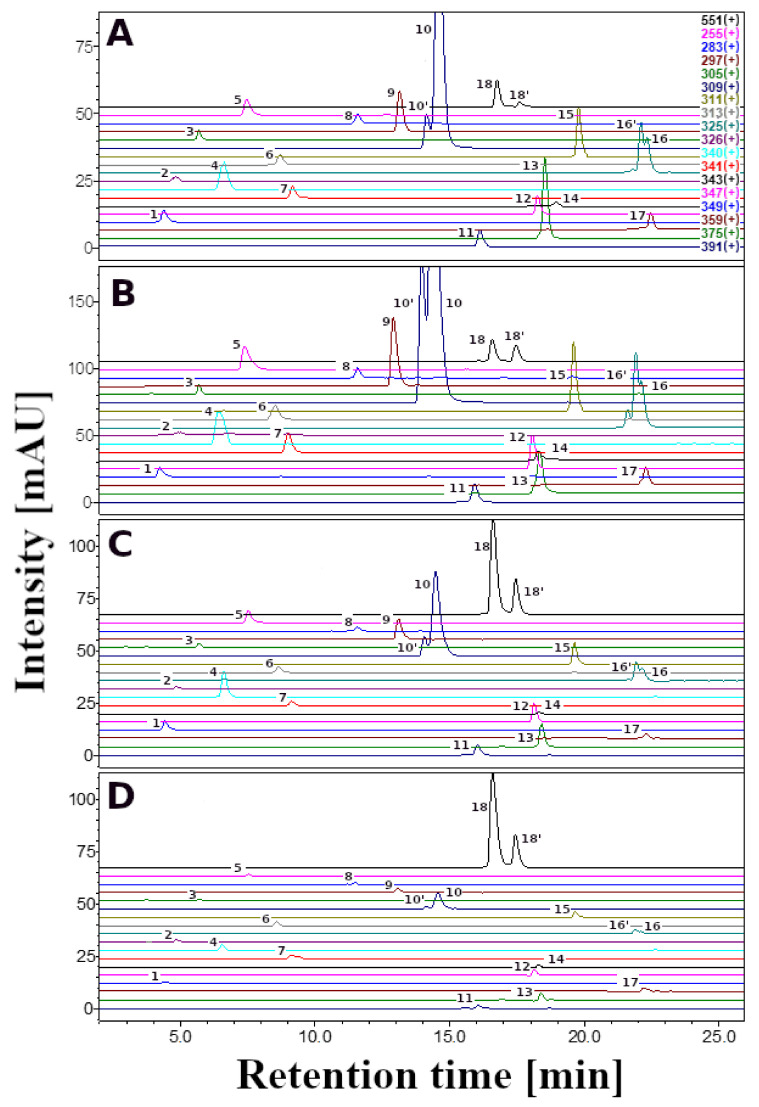
Selected-ion monitoring chromatogram (ESI-MS) in positive ion mode for betalains from *P. grandiflora*: (**A**) yellow; (**B**) orange; (**C**) red; (**D**) purple.

The sample preparation and extraction method adopted in the present study allowed us to obtain betalain derivatives—betacyanins and betaxanthins (Table 1 and Table 2, Figure 1 and Figure 2). The highest total concentration of betalains was noticed in the dried extract of the orange *P. grandiflora* (1132 mg/100 g DE). The highest concentration of betaxanthins was noticed in the dried extract of the orange variety (982 mg/100 g DE), while the red one accumulated the lowest betaxanthin content (162 mg/100 g DE). A similar betaxanthin concentration was noticed in the yellow and purple flower dried extracts of *P. grandiflora* (417 and 323 mg/100 g DE, respectively). A betacyanin-rich source was revealed to be the purple variety of *P. grandiflora* dried extract (650 mg/100 g DE). A significantly lower betacyanin concentration was noticed in the orange and red flower dried extracts (150 and 144 mg/100 g DE, respectively). In the case of the yellow *P. grandiflora* dried extract, the lowest concentration of betacyanins was noticed (64.3 mg/100 g DE) (Table 1). 

The HPLC-DAD-ESI/MS analysis of betaxanthins in *P. grandiflora* flower dried extracts revealed identical profiles in yellow, orange, red and purple varieties, however, the profile of betaxanthins is more complex than reported previously [15]. Here, we report the identification of fifteen new betaxanthins in *P. grandiflora* (Table 1). Proline-Bx (indicaxanthin) (**11**), which is the major pigment in cactus pear [35] and has not been identified in previous *P. grandiflora* extracts, was revealed to be dominant in the studied *P. grandiflora* varieties (46.0–330 mg/100 g DE). Moreover, *P. grandiflora* dried extracts, might be a good source of glutamine-Bx (**4**), ethanolamine-Bx (**5**), glutamic acid-Bx (**7**), *γ*-aminobutyric acid-Bx (**9**), proline-IBx (indicaxanthin) (**10**), dopa-Bx (**12**), dopamine-Bx (**13**), Tyrosine-Bx (**14**), Valine-Bx (**16**), (iso)leucine-Bx (**17**/**18**) and phenylalanine (**19**) (Table 1). Studied *P. grandiflora* dried extracts might be a relatively good source of betanin (**20**) and isobetanin (**21**), especially the purple variety (484 and 166 mg/100 g DE, respectively) (Table 1).

The presence of betalains may contribute to the biological effect of the tested plants, however, the effect of other compounds in the extract cannot be excluded. Therefore, a phytochemical complex profile was determined for the studied plants by the HPLC-DAD-ESI-HRMS/MS method. The analysis revealed the presence of 71 compounds, including nineteen betaxanthins, two betacyanins, seven organic acids and their derivatives, three amino acids and their derivatives, three hydroxybenzoic acids and their derivatives, eleven hydroxycinnamic acids and their derivatives, one hydrolisable tannin and its derivatives, ten fatty acid and their derivatives, one flavone and its derivatives and fourteen flavonoid and their derivatives (Table 2). It is worth noting that all identified metabolites were included in the yellow, orange, red and purple flowers of *P. grandiflora*. For all compounds, the correlation coefficients between the identified compounds (absolute peak area of each assigned peak from the chromatograms) and the bioactivity results were calculated for all studied extracts (Table 3 and Table 4). The phytochemical profiles have been determined for the first time in *P. grandiflora*.

### 3.2. Antioxidant Activity

Measurements of antioxidant activity can be related to the capacity of extracts to directly transfer hydrogen to a radical (ABTS or DPPH) and to donate their electrons (FRAP) [51]. Therefore, the measurement of antioxidant activity cannot be determined by a single method, and more than one type of assay must be performed to take into account the various modes of antioxidant activity. 

The antioxidant and free-radical scavenging potential of the extracts of the yellow, orange, red and purple varieties of *P. grandiflora* were evaluated by several in vitro cell-free assays ABTS, DPPH and FRAP. Table 3 shows the values of the antioxidant potential of tested extracts expressed as mM trolox equivalent per gram of dry extract weight (DE). 

In the ABTS assay, the values were in the range of 0.079 and 0.176 mM TE/g DE, which represents a variation of approximately 2-fold. In this assay, the red variety of *P. grandiflora* showed the highest antioxidant activity (0.176 mM TE/g DE), followed by the purple (0.135 mM TE/g DE), orange (0.112 mM TE/g DE) and yellow varieties (0.079 mM TE/g DE). 

In the DPPH assay, the values were in the range of 0.078 and 0.174 mM TE/g DE, which means that there was the same 2-fold variability as was in the ABTS assay. In this test, the red variety of *P. grandiflora* also possessed the highest antioxidant activity (0.174 mM TE/g DE), followed by the purple (0.125 mM TE/g DE), orange (0.111 mM TE/g DE) and yellow varieties (0.078 mM TE/g DE). 

FRAP values varied from 0.066 to 0.160 mM TE/g DE, which represents a higher variation than that seen in the ABTS and DPPH assays, of 2.5-fold more. In this assay, the red variety of *P. grandiflora* also showed the highest antioxidant activity (0.160 mM TE/g DE), followed by the purple (0.077 mM TE/g DE) and the orange (0.067 mM TE/g DE). The yellow variety possessed the lowest antioxidant potential (0.066 mM TE/g DE) as it also did for the ABTS and DPPH tests. 

The obtained values of antioxidant capacity from the ABTS and DPPH assays are comparable. In contrast, in some cases, the antioxidant activity values obtained by the FRAP assay differ significantly from those obtained by the DPPH and ABTS assays, which may be due to the fact that some compounds, such as phenolic acids, tend to react very slowly with the parent complex, requiring a longer reaction time [52]. In conclusion, the red variety of *P. grandiflora* showed the highest antioxidant activity in all the assays. For the other varieties, their activity was decreased in a sequence of purple > orange > yellow. 

Ascorbic acid is very popular due to its antioxidant properties, so it was used as a reference compound. The investigated extracts of *P. grandiflora* showed much lower antioxidant activity than pure ascorbic acid. Nevertheless, it is hard to compare the complex matrices of the extracts to the pure compound. The obtained results of the antioxidant activity of ascorbic acid measured by the ABTS and DPPH methods were similar (7.19 and 8.07 mM TE/g DE, respectively). On the other hand, the result obtained by the FRAP method (3.36 mM TE/g DE) differs significantly from the others, which may be due to the fact that the presence of iron (III) ions in the FRAP reagent may significantly intensify the oxidation of ascorbic acid.

### 3.3. Correlation between Antioxidant Activity and Phytochemical Composition

The antioxidant activity results (mM TE/g DE) of the extracts of *P. grandiflora* obtained from the ABTS, FRAP and DPPH assays were correlated with their metabolite composition (absolute peak area of each assigned peak from the chromatograms) and are presented in Table 3 using the correlation coefficients (R). There was no significant correlation between betalains (betaxanthins and betacyanins) and antioxidant activities (*p* < 0.05). The exception is that betanin belonged to the betacyanin group, which showed significant and weak correlation with the antioxidant activity when measured only with the ABTS and DPPH assays (R = 0.273 and 0.170, respectively). All identified betaxanthins showed a negative correlation, which indicates that other compounds might be responsible for the antioxidant potential of the extracts, for example from the polyphenol group. The high antioxidant activity of betanin is well-documented [53]. The bioavailability of betanin is estimated as rather low [54].

The group of compounds from organic acids with derivatives showed a negative or weak (R = 0–0.3) correlation with antioxidant activity. An exception to this is seen in malic acid, which showed a very strong (R = 0.7–1.0) and significant correlation against antioxidant potential determined by the ABTS and DPPH methods (R = 0.878 and 0.824, respectively) and a strong correlation (R = 0.5–0.7) against activity determined by the FRAP method, only (R = 0.652). The antioxidant activity of malic acid was confirmed previously [55]. Malic acid is considered as safe when it is at low concentrations. The oral LD_50_ value of malic acid in rabbits ranged from 3–5 g/kg [56]. *N*-(carboxyacetyl) phenylalanine from the group containing the amino acids with derivatives had a significant and very strong correlation with the antioxidant activity measured by all the ABTS, FRAP and DPPH methods (R = 0.977. 0.958 and 0.993, respectively). Tryptophan showed a negative or weak correlation with regard to antioxidant activity. *N*-Benzoylaspartic acid had a significant and moderate (R = 0.3–0.5) correlation with antioxidant potential as determined by the FRAP assay (R = 0.428). Previous research showed, that amino acids have the ability to prevent or attenuate oxidative processes [57]. Compounds belonging to the group of hydroxybenzoic acids with derivatives showed a negative or weak correlation for antioxidant capacity, with the exception of hydroxybenzoic acid. Hydroxybenzoic acid is characterized by a strong correlation with the activity as determined by the ABTS method (R = 0.530) and a moderate correlation with the activity as determined by the DPPH method (R = 0.440). Antioxidant activity of hydroxybenzoic acid was previously confirmed [58]. As many as eleven compounds from the group containing the hydroxycinnamic acids with derivatives were identified, but only three of them showed a positive correlation with antioxidant activity. It is worth noting that two metabolites (feruloylquinic acid and rosmarinic acid) showed a very strong correlation against activity as determined by all methods (ABTS, FRAP and DPPH). Whereas it was observed that ferulic acid hexose I was characterized by a weak correlation (R = 0.076). Hydroxycinnamic acids are described as potent antioxidants [59]. A previous study has confirmed that rosmarinic acid possesses good antioxidant activity [60]. It is worth noting that among the group of hydrolysable tannins and their derivatives, only galloyl hexoside was identified, which showed a significant and very strong correlation with the antioxidant potential (R = 0.775, 0.919 and 0.799, respectively) as determined by all methods (ABTS, FRAP and DPPH). Tannins are substances with strong antioxidant and antimicrobial activities. The mechanisms of the bioavailability of tannins is poorly studied [61]. It was observed that all metabolites in the fatty acids and their derivatives group had a significant and positive correlation with antioxidant capacity, except for tuberonic acid, towards the activity as measured by the FRAP method. Trihydroxyoctadecadienoic acid, hydroperoxyoctadecadienoic acid and hydroxyoctadecatrienoic acid I are characterized by a significant and very strong correlation with antioxidant activity as determined by all methods (ABTS, FRAP and DPPH). Tuberonic acid hexoside and dihydroxyhexadecanoic acid showed a significant and strong correlation with the activity as determined by the ABTS and DPPH methods, while when antioxidant activity was determined by the FRAP method, it showed a weak (R = 0.160) and moderate (R = 0.396) correlation, respectively. In this group, the remaining metabolites showed a moderate or weak correlation with the antioxidant potential. Selected fatty acids are described as highly bioavailable compounds [62]. Moreover, they are indicated as antioxidants and antimicrobials [63]. From the group containing the flavones and their derivatives, only luteolin-6,8-*C*-dihexose was identified, which showed a significant and moderate antioxidant capacity when measured by all methods (ABTS, FRAP and DPPH). Fourteen metabolites were identified from the group containing the flavonoids and their derivatives which showed significant effects on antioxidant activity. As many as half of them (quercetin-*O*-hexoside I, quercetin-*O*-hexoside II, luteolin, naringenin, apigenin II, cirsimaritin and sorbifolin) showed significant a positive and very strong correlation with antioxidant activity as measured by different assays (ABTS or FRAP or DPPH);in this group, five compounds (quercetin-*O*-hexoside II, luteolin-7-*O*-rutinoside II, naringenin, apigenin II and sorbifolin) also showed significant a positive and strong correlation with activity as measured by different assays (ABTS or FRAP or DPPH). Luteolin-7-*O*-rutinoside I, luteolin-7-*O*-rutinoside III and luteolin-*O*-hexoside had a negative correlation with antioxidant activity as measured by all methods (ABTS, FRAP and DPPH), whereas apigenin I showed only a negative correlation with antioxidant activity when measured by FRAP. Flavonoids and flavone act in plants as antioxidants [64]. Their bioavailability is generally low but efforts are being made to improve this parameter [65]. In conclusion, significant impacts on the antioxidant activity of *P. grandiflora* extracts were shown by the metabolites belonging to the groups containing flavonoids and their derivatives, fatty acids and their derivatives and hydrolisable tannins and their derivatives, as well as individual compounds from other groups such as malic acid, *N*-(carboxyacetyl) phenylalanine, feruloylquinic acid and rosmarinic acid. In contrast, betalains showed no significant effect on antioxidant properties. These results suggest that the antioxidant capacity of *P. grandiflora* results to a much greater extent from the presence of phenolic compounds and flavonoids than it does from betalains. The antioxidant potential of individual, pure betalains cannot be excluded, which has been confirmed in numerous literatures [16,17,18,19,20]. The extract is a complex mixture that may contain compounds that enhance or inhibit biologically active compounds.

### 3.4. Antimicrobial Activity

The minimum inhibitory concentrations of the tested extracts obtained from flowers of *Portulaca grandiflora* measured during the antimicrobial test that used the broth microdilution method are presented in Table 4.

These four extracts (yellow, orange, red and purple) showed different activity levels against bacteria (MIC = 4–16 mg/mL) and yeasts (MIC = 32 mg/mL), suggesting that bacterial strains are more susceptible than yeasts strains. The highest activity was observed against *M. luteus* ATCC 10240 and *B. subtilis* ATCC 6633 with MIC = 4 mg/mL for yellow and orange extracts, respectively. It was observed that extracts from the yellow and orange flowers of *P. grandiflora* flowers also showed very good activity against *B. cereus* ATCC 10876 and staphylococci with MIC = 8 mg/mL. 

There were some differences between the antibacterial activity of extracts from yellow and orange flowers, and extracts from red and purple flowers of *P. grandiflora*. Generally, extracts from yellow and orange flowers were more active against Gram-positive bacteria than extracts from red and purple flowers, while extracts from red and purple flowers were slightly more active against Gram-negative bacteria. All the extracts had the same activity against yeasts with MIC and MFC values of 32 mg/mL.

The MIC for the reference antimicrobial substances were the following: the MIC of vancomycin for *S. aureus* ATCC 29213 was 1 μg/mL, the MIC of ciprofloxacin for *E. coli* ATCC 25922 was 0.5 μg/mL and the MIC of fluconazole for *C. albicans* ATCC 10231 was 1 μg/mL. In contrast, the MIC for the extracts from yellow, orange, red, and purple flowers of *P. grandiflora* against *S. aureus* ATCC 29213 was 8–32 mg/mL, for *E. coli* ATCC 25922 it was 16 mg/mL and against *C. albicans* ATCC 10231 it was 32 mg/mL. The reference substances definitely show better antimicrobial activity than the tested extracts did. 

In this study, the MBC and MFC that complement the MIC was assessed. The MBC or MFC was identified by determining the lowest concentration of extracts that kill 99.9% bacteria or yeasts over a fixed, somewhat extended period, such as 18 ± 2 h, under specific conditions. The MBC and MFC studies were used to assess the potency of the extracts. The extracts were usually regarded as bactericidal and fungicidal if the MBC or MFC was no more than four times that of the MIC (the MBC/MIC or MFC/MIC ratio is ≤4) [66]. It was found that all tested extracts possessed a bactericidal and fungicidal effect showed by that fact that MBC/MIC = 1–4 and MFC/MIC = 1, respectively.

The research on the antimicrobial activity of *P. grandiflora* and *P. oleracea* extracts has so far been determined using the agar well diffusion method whereby zones of growth inhibition in diameter [mm] were defined [1,67]. In this study, the results of the activity of *P. grandiflora* extracts were presented for the first time, determining the lowest concentration inhibiting the growth of microorganisms as MICs values as per the broth microdilution method. The presented data suggest that extracts obtained from the yellow, orange, red, and purple flowers obtained from *P. grandiflora* may be regarded as a promising source of natural compounds with biocidal. It is worth noting that there is extensive antibacterial activity (MIC = 8 mg/mL) of extracts from yellow and orange flowers of *P. grandiflora* against bacteria causing food poisoning such as *Staphylococcus aureus* and *Bacillus cereus*.

### 3.5. Correlation between Antimicrobial Activity and Phytochemical Composition

The results of the study of the microbial activity (MIC values) of extracts of *P. grandiflora* were correlated with their metabolite composition (absolute peak area of each assigned peak from the chromatograms) and presented in Table 4 with correlation coefficients (R). The lower the MIC value is, then the better the antimicrobial activity is. Therefore, a positive effect on antimicrobial activity was described as the negative value of the correlation coefficient, R (negative correlation).

Betaxanthins had a greater effect on microbial activity than betacyanins did. All identified betaxanthins exhibited a negative correlation with the antibacterial effect against all tested strains of Gram-positive bacteria, except for asparagine-Bx, histamine-Bx, dopa-Bx, dopamine-Bx and tyrosine-Bx. In contrast, all betaxanthins showed a positive correlation with the antibacterial effect against all Gram-negative bacterial strains, except for histidine-Bx, tyrosine-Bx and methionine-Bx. All betaxanthins showed a negative correlation with the antifungal effect against *C. albicans* ATCC 10231 with the exception of asparagine-Bx, histamine-Bx, dopa-Bx, dopamine-Bx and tyrosine-Bx. On the other hand, all betaxanthins had a positive correlation with the antifungal effect against *C. glabrata* ATCC 90030 with the exception of histidine-Bx and glutamine-Bx. It is worth noting that the betacyanins (betanin and isobetanin) exhibited the only negative correlation with the antibacterial effect against two strains of Gram-negative bacteria, *E. coli* ATCC 25922 and *S. typhimurium* ATCC 14028. The antimicrobial activity of purified betaxanthins has been never studied before. This research on the antimicrobial activity of betacyanins confirmed their action [25].

From the group containing organic acids and their derivatives, malic acid, 2-hydroxyquinoline-3-carboxylic acid, benzoic acid and carnosic acid showed a positive correlation with the antibacterial effect against all strains of Gram-positive bacteria, and feruloylmalic acid also did so against all strains of Gram-negative bacteria. Those which showed a positive correlation with the antifungal effect against all yeast strains that were tested were 2-Hydroxyquinoline-3-carboxylic acid, benzoic acid and feruloylmalic acid. However, gluconic acid, citric acid and feruloylmalic acid exhibited a negative correlation with the antibacterial effect against almost all tested strains of Gram-positive bacteria. Gluconic acid and carnosic acid showed a negative correlation with the antibacterial effect against all strains of Gram-negative bacteria, whereas malic acid, gluconic acid, citric acid, 2-hydroxyquinoline-3-carboxylic acid and benzoic acid also did so against almost all strains of Gram-negative bacteria. It is worth emphasizing that only gluconic acid from the group containing the organic acids and their derivatives proved to have a negative correlation with the antifungal effect against all yeast strains. Interestingly, research on betalains shows that organic acids may act as antimicrobial substances as well as enhancing the antimicrobial activity of selected derivatives, including organic acids [25].

From the group containing the amino acids and their derivatives, only *N*-benzoylaspartic acid showed a negative correlation with the antibacterial and antifungal effect toward all tested microorganisms. On the other hand, the other metabolites in this group (tryptophan and *N*-(carboxyacetyl) phenylalanine) had a positive correlation with the antimicrobial effect against all Gram-positive bacteria and also did so with the antifungal activity against all yeast strains. It should be noted that all the identified metabolites from the group containing the amino acids and their derivatives influenced the antimicrobial activity against all strains of Gram-negative bacteria, except for *N*-(carboxyacetyl) phenylalanine, which showed a positive correlation against *P. aeruginosa* ATCC 27853. Amino acids arouse the interest due to their anticancer or neuroprotective effects, but they also enact antimicrobial activities [68]. Metabolites from the group containing the hydroxybenzoic acids and their derivatives exhibited varying effects on antimicrobial activity. Vanillic acid hexoside was the only one that showed a negative correlation with the antibacterial effect against all microorganisms, except for *E. coli* ATCC 25922. On the other hand, hydroxybenzoic acid was the only one to show a positive correlation with the antimicrobial effect against all microorganisms, except for *E. coli* ATCC 25922 and *S. typhimurium* ATCC 14028. 

From the group containing the hydroxycinnamic acids and their derivatives, as many as seven metabolites (caffeic acid hexoside, ferulic acid hexose I, chlorogenic acid I, ferulic acid hexose II, 4-*p*-coumaroylquinic acid I, 1-*O*-sinapoyl-beta-*D*-glucose and *p*-coumaric acid) exhibited a negative correlation with the antibacterial effect against all strains of Gram-positive bacteria. On the contrary, chlorogenic acid II and feruloylquinic acid had a positive correlation with the antibacterial effect against all Gram-positive bacteria. It should be noted that ferulic acid hexose I, chlorogenic acid I and ferulic acid hexose II had a negative correlation with the antifungal effect against all yeast strains. Antimicrobial activity was confirmed, previously, for hydroxybenzoic and hydroxycinnamic acids [69]. 

Galloyl hexoside, belonging to the group containing the hydrolysable tannins and their derivatives, showed a negative correlation with the antimicrobial effect against all strains of Gram-negative bacteria and yeasts. On the other hand, it had a differentiated effect on the antibacterial activity against Gram-negative bacteria.

All tested metabolites belonging to the group containing the fatty acids and their derivatives were characterized by a positive influence on the microbiological activity against all Gram-positive bacteria, except for tuberonic acid hexoside. Nevertheless, a negative correlation of all metabolites from this group was also observed for the antimicrobial activity against two strains of Gram-negative bacteria, *E. coli* ATCC 25922 and *S. typhimurium* ATCC 14028, with the exception of tuberonic acid hexoside. 

Luteolin-6,8-*C*-dihexose, from the flavones and their derivatives group, had a negative correlation with the antimicrobial effect against all strains of Gram-positive bacteria and yeasts, and a positive correlation against all strains of Gram-negative bacteria.

From the group containing the flavonoids and their derivatives, luteolin-7-*O*-rutinoside I, luteolin-7-*O*-rutinoside III, genistein-4′-*O*-glucoside and apigenin I exhibited a negative e correlation with the antibacterial effect against all strains of Gram-positive bacteria; these were quercetin-*O*-hexoside I, luteolin-*O*-hexoside, luteolin, naringenin, apigenin II, sorbifolin and kaempferol against Gram-negative bacteria. Luteolin-7-*O*-rutinoside I, luteolin-7-*O*-rutinoside II, genistein-4′-*O*-glucoside, naringenin and apigenin II showed a negative correlation with the antifungal effect against all yeast strains. Flavones and flavonoids are well known antibacterial and antifungal agents against a ranged panel of microorganisms [70].

In summary, the greatest effect on the antibacterial activity against Gram-positive bacteria was shown by the metabolites belonging to the groups of betaxanthins, hydroxybenzoic acids with derivatives and flavones with derivatives, as well as individual compounds from other groups, such as gluconic acid, citric acid, *N*-benzoylaspartic acid, vanillic acid hexoside, luteolin-7-*O*-rutinoside I, luteolin-7-*O*-rutinoside III, genistein-4′-*O*-glucoside and apigenin I. On the other hand, the metabolites belonging to groups containing the betacyanins, amino acids and their derivatives, hydrolysable tannins and their derivatives, fatty acids and their derivatives and flavonoids and their derivatives had the greatest influence on antibacterial activity against Gram-negative bacteria. The metabolites belonging to the groups and derivatives of hydrolysable tannins, flavones and flavonoids showed the greatest antifungal activity against yeast strains. These results suggest that the better antimicrobial activity of the *P. grandiflora* extracts is usually associated with the presence of a specific group of ingredients. 

### 3.6. Cytotoxicity Evaluation

The cytotoxicity evaluation of extracts obtained from *P. grandiflora* flowers was carried out using a microculture tetrazolium assay (MTT). The MTT assay measures the ability of cellular dehydrogenases to reduce a yellow substrate (3-(4,5-dimethylthiazol-2-yl)-2,5-diphenyltetrazolium bromide) to a violet formasane product, which is insoluble in water and requires an appropriate solvent (a mixture of sodium dodecyl sulphate, dimethylformamide and PBS) to dissolve the formasane crystals. Since the cellular dehydrogenases remain biologically active only in viable, metabolically active cells, the level of enzyme activity can be used to measure their viability.

**Figure 3:**
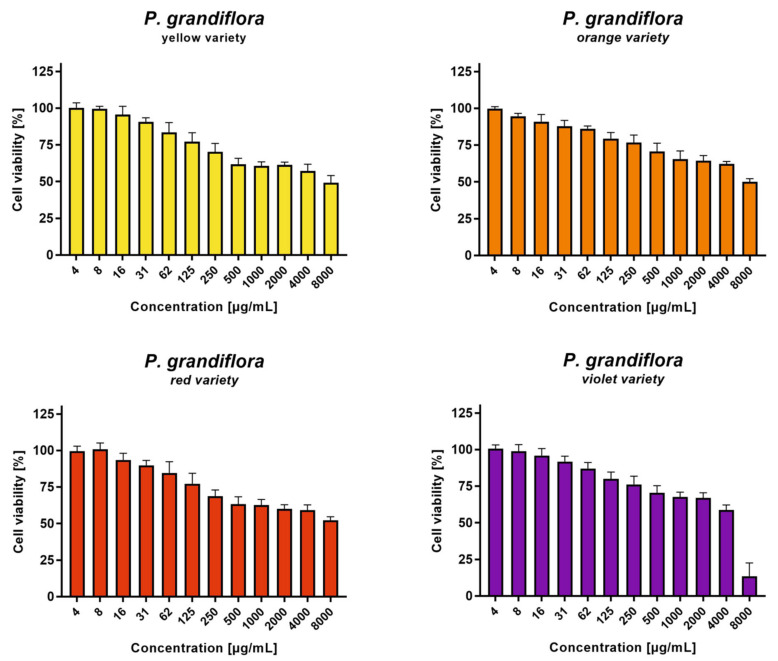
Cytotoxicity of *P. grandiflora* extracts towards VERO cells.

The result of the cytotoxicity assessment towards normal–VERO cells is presented in Figure 3. In the case of yellow, orange and red varieties of *P. grandiflora*, the highest tested concentration of 8000 µg/mL reduced the viability of the VERO cells by approx. 50%, as compared with the control cells. The purple variety of *P. grandiflora* showed higher toxicity, reducing the cellular viability by approx. 85% at 8000 µg/mL. The criteria of the plant extract cytotoxicity evaluation, based on the guidelines set by the National Cancer Institute (NCI) [71] and the published literature [72], indicate that if the CC_50_ (50% cytotoxic concentration) of the tested extract is above 500 µg/mL, then the extract can be classified as not cytotoxic. In the case of *P. grandiflora*, the CC_50_ values were not calculated because most extracts did not reduce the viability by more than 50% at 8000 µg/mL. This allows for a conclusion that *P. grandiflora* flower extracts are deprived of any significant cytotoxicity towards the VERO cell line. 

It was also necessary to evaluate the cytotoxicity of *P. grandiflora* extracts towards the VERO cells after 72 h incubation because a similar incubation time is required in the studies of antiviral activity. Moreover, the concentrations of the samples tested as potential antivirals cannot show significant cytotoxicity on the cells used for virus propagation, that is, they must not decrease the cellular viability by more than 10%. It was found that all tested *P. grandiflora* extracts were nontoxic to the VERO cells in the concentration of above 62 µg/mL, and this concentration was selected as it was the highest used in antiviral studies. 

**Figure 4:**
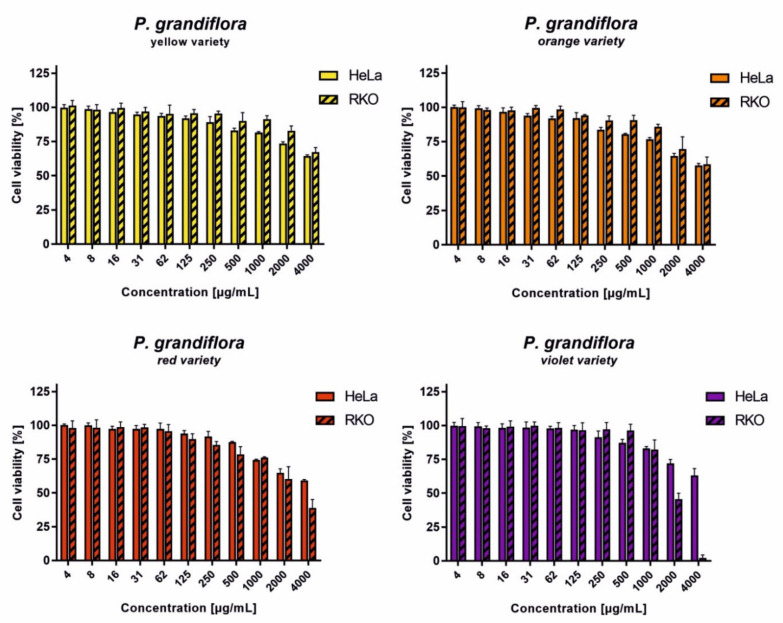
The dose-response activity of *P. grandiflora* extracts on cancer cell lines after 24 h incubation.

The evaluation of the anticancer potential of *P. grandiflora* extracts was performed after 24 h incubation towards HeLa (cervical adenocarcinoma) and RKO (colon carcinoma) cells, derived from a cervical adenocarcinoma and colon cancer, respectively. The results are presented in Figure 4. In the case of the yellow and orange varieties, at 4000 µg/mL the cellular viability was above 50% for both cancer cell lines. A similar effect was observed for red and purple varieties on the HeLa cells. Whereas, for the red and purple varieties, noticeably higher cytotoxicity was found on the RKO cells. The highest anticancer potential was observed for the *P. grandiflora* purple variety extract towards the RKO cells, with less than 5% cellular viability at 4000 µg/mL, and the CC_50_ of 1794 ± 74.95 µg/mL. It is worth noting, the purple variety of *P. grandiflora* was the richest source of betacyanins when compared to the other varieties (650 mg/100 g DE). This suggests that the cytotoxicity of the extracts might be affected by the concentration of betalains.

### 3.7. Antiviral Activity

**Figure 5:**
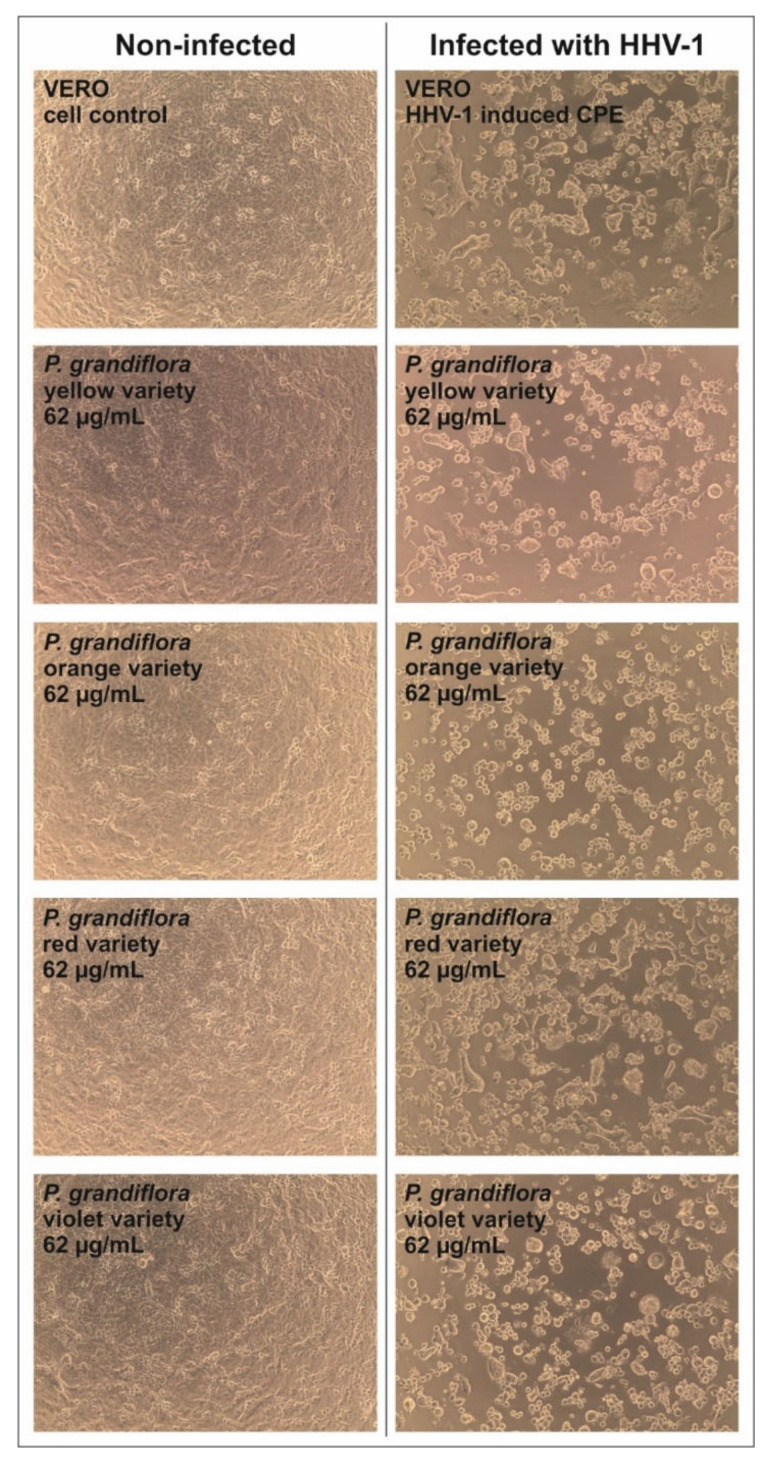
The effect of *P. grandiflora* extracts on HHV-1-related CPE.

**Figure 6:**
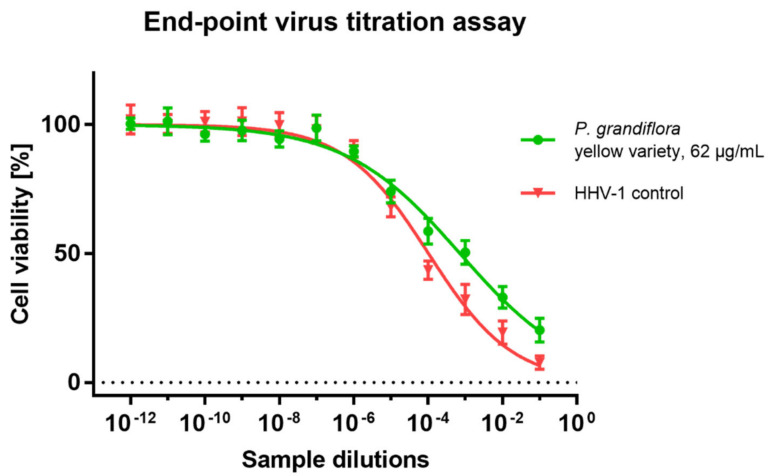
The end-point dilution assay of HHV-1 infectious titer in *P. grandiflora* yellow-variety extract-treated infected cells.

**Figure 7:**
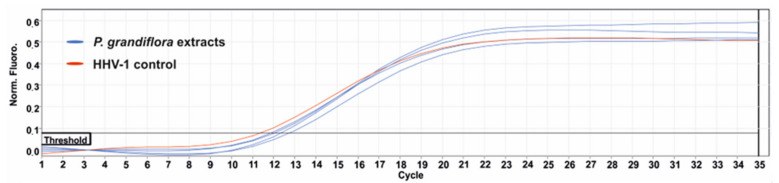
The qPCR amplification curves used for the measurement of HHV-1 viral load.

The results of the influence of *P. grandiflora* extracts on the formation of CPE in the HHV-1 infected VERO cells are presented in Figure 5. Based on the observed results, it can be concluded that none of the tested extracts showed any noticeable effect on HHV-1-induced CPE. The evaluation of the HHV-1 infectious titer in the extract-treated samples (Table 5 and Figure 6) showed that *P. grandiflora* extracts decrease the infectious titer by 0.2–0.69 log (Δlog CCID_50_/mL), which confirms the lack of any significant effect on HHV-1 replication. To further evaluate the effect of *P. grandiflora* extracts on the replication of HHV-1 in infected the VERO cells, we have measured the viral load in DNA isolates from samples collected from antiviral assays. The Real-Time PCR quantification of the HHV-1 load was performed in relation to a calibration curve which was previously titrated using a commercially available diagnostic kit. As can be concluded from Table 5, the *P. grandiflora* extracts decreased the HHV-1 viral load by 0.17–0.44 log (HHV-1 copies/µL), which corresponds with the results observed for the end-point dilution assay. The amplification curves are presented in Figure 7. A postamplification melt analysis showed the presence of a specific product with a melting temperature of 85.5 °C in all tested isolates. 

## 4. Conclusions

This is the first study investigating the accurate phytochemicals profile, including betalains as well as antioxidant, antimicrobial and antiviral activity and cytotoxicity of betalain-rich extracts from flowers of *Portulaca grandiflora* L. Moreover, this is the first study evaluating the correlation between the phytochemical profile and the biological activity of *P. grandiflora*.

*P. grandiflora* showed good free radicals scavenging potential as well as inhibitory activity against a wide range of Gram-positive and Gram-negative bacteria and fungi. The antioxidant activity found in all studied extracts allow us to postulate that betalains are not responsible for the antioxidant power of *P. grandiflora*. A significant impact on the antioxidant activity of *P. grandiflora* extracts was shown by metabolites belonging to the group of flavonoids and their derivatives, fatty acids and their derivatives and hydrolisable tannins and their derivatives, as well as individual compounds from other groups such as malic acid, *N*-(carboxyacetyl) phenylalanine, feruloylquinic acid and rosmarinic acid. The presence of more compounds in the *P. grandiflora* extracts could weaken the action of the betalains because the antioxidant potential of pure betalains has been previously confirmed.

Metabolites belonging to the group containing the betaxanthins, hydroxybenzoic acids and their derivatives and flavones and their derivatives, as well as individual compounds from other groups, such as gluconic acid, citric acid, *N*-benzoylaspartic acid, vanillic acid hexoside, luteolin-7-*O*-rutinoside I, luteolin-7-*O*-rutinoside III, genistein-4′-*O*-glucoside and apigenin I proved to be the most powerful compounds against the Gram-positive bacteria. Betacyanin and amino acids and their derivatives, hydrolysable tannins and their derivatives, fatty acids and their derivatives, and flavonoids and their derivatives had the greatest influence on antibacterial activity against Gram-negative bacteria. The metabolites belonging to hydrolysable tannins and their derivatives, flavones and their derivatives and flavonoid and their derivatives showed the greatest antifungal activity against yeast strains.

Our results show that *P. grandiflora* extracts are not cytotoxic to normal VERO cells. Moreover, an antiviral assessment showed that the *P. grandiflora* extracts have no significant antiviral activity towards Human Herpesvirus type 1.

Research on correlation between the phytochemical profile and the biological activity of the plant allows us to select the compounds that may show a biological effect. However, the complexity of the plant matrix does not confirm the inactivity of a compound.

## Figures and Tables

**Table 1:** Content of individual betalains and total betalains identified by HPLC-DAD-ESI-MS in the flowers of yellow, orange, red and purple *P. grandiflora*.

No.	Betalains	Content of Individual Betalains (mg/100 g DE) **				
		Yellow	Orange	Red	Purple	Fisher’ LSD
	**Betaxanthins**					
1	Histidine-Bx	7.7 ± 0.25 ^d^	7.5 ± 0.18 ^c^	5.6 ± 0.28 ^b^	4.4 ± 0.18 ^a^	0.099
2	Asparagine-Bx	1.8 ± 0.07 ^b^	4.1 ± 0.17 ^c^	0.91 ± 0.05 ^a^	6.6 ± 0.26 ^d^	0.079
3	Histamine-Bx *	5.4 ± 0.21 ^b^	10.7 ± 0.30 ^d^	2.5 ± 0.13 ^a^	7.1 ± 0.29 ^c^	0.076
4	Glutamine-Bx	15.4 ± 0.23 ^c^	33.6 ± 0.34 ^d^	14.5 ± 0.35 ^b^	7.1 ± 0.29 ^a^	0.074
5	Ethanolamine-Bx	9.3 ± 0.22 ^b^	23.6 ± 0.24 ^d^	3.4 ± 0.16 ^a^	11.0 ± 0.23 ^c^	0.136
6	Threonine-Bx	6.2 ± 0.17 ^c^	14.5 ± 0.29 ^d^	3.2 ± 0.14 ^a^	6.0 ± 0.24 ^b^	0.101
7	Glutamic acid-Bx	6.9 ± 0.22 ^c^	20.0 ± 0.40 ^d^	2.3 ± 0.10 ^a^	3.6 ± 0.14 ^b^	0.095
8	Alanine-Bx	5.8 ± 0.22 ^c^	9.6 ± 0.39 ^d^	2.5 ± 0.12 ^a^	4.4 ± 0.18 ^b^	0.090
9	*γ*-Aminobutyric acid-Bx	23.1 ± 0.32 ^c^	71.7 ± 0.93 ^d^	11.2 ± 0.28 ^a^	19.2 ± 0.58 ^b^	0.094
10	Proline-IBx	19.3 ± 0.39 ^c^	137 ± 5.50 ^d^	11.4 ± 0.28 ^b^	11.0 ± 0.33 ^a^	0.122
11	Proline-Bx	154 ± 1.50 ^c^	330 ± 9.90 ^d^	46.0 ± 0.83 ^a^	79.9 ± 1.40 ^b^	0.119
12	Dopa-Bx	10.0 ± 0.19 ^b^	18.9 ± 0.68 ^d^	5.8 ± 0.27 ^a^	16.5 ± 0.33 ^c^	0.086
13	Dopamine-Bx	10.8 ± 0.19 ^b^	34.4 ± 0.69 ^d^	9.8 ± 0.27 ^a^	32.9 ± 0.66 ^c^	0.061
14	Tyrosine-Bx	46.2 ± 0.46 ^d^	43.8 ± 0.88 ^c^	12.5 ± 0.50 ^a^	38.4 ± 0.79 ^b^	0.049
15	Methionine-Bx	4.4 ± 0.18 ^b^	5.5 ± 0.28 ^c^	1.1 ± 0.06 ^a^	1.1 ± 0.05 ^a^	0.027
16	Valine-Bx	29.0 ± 0.43 ^b^	71.5 ± 0.89 ^d^	12.0 ± 0.24 ^a^	30.2 ± 0.91 ^c^	0.091
17	Isoleucine-Bx	29.3 ± 0.38 ^c^	77.3 ± 0.98 ^d^	10.3 ± 0.21 ^a^	23.7 ± 0.71 ^b^	0.015
18	Leucine-Bx	20.8 ± 0.37 ^c^	48.2 ± 0.77 ^d^	6.8 ± 0.34 ^a^	13.7 ± 0.41 ^b^	0.049
19	Phenylalanine-Bx	11.9 ± 0.14 ^c^	19.2 ± 0.31 ^d^	0.28 ± 0.01 ^a^	6.0 ± 0.33 ^b^	0.049
	**Betacyanins**					
20	Betanin	54.3 ± 0.04 ^a^	82.8 ± 0.02 ^b^	109 ± 0.01 ^c^	484 ± 0.01 ^d^	0.090
21	Isobetanin	10.7 ± 0.02 ^a^	61.6 ± 0.01 ^c^	40.3 ± 0.02 ^b^	166 ± 0.02 ^d^	0.076
**Total betaxanthin mass (mg/100 g DE)**		417 ± 5.4 ^c^	982 ± 12.8 ^d^	162 ± 3.4 ^a^	323 ± 6.1 ^b^	0.060
**Total betacyanin mass (mg/100 g DE)**		64.3 ± 0.99 ^a^	144 ± 5.8 ^b^	150 ± 5.7 ^c^	650 ± 6.9 ^d^	0.012

* Tentatively identified; ** Mass in 100 g of dry extract (DE) flowers of *P. grandiflora*. Data are expressed as mean ± standard deviation (*n* = 3). Values in each row having the same letter are not significantly different (*p* > 0.05).

**Table 2:** High-resolution mass spectrometric data obtained by HPLC-DAD-ESI-HRMS/MS analysis of metabolites in the flowers of yellow, orange, red and purple *P. grandiflora*, analysed in negative and positive ionization modes.

No.	Metabolite	Molecular Formula	t_R_ [min]	*m*/*z* [M+H]^+^_exp_	*m*/*z* [M+H]^+^_caled_	Δ [ppm]	*m*/*z* from MS^2^ of [M+H]^+^	References
	***Betaxanthins***							
1	Histidine-Bx (muscaaurin VII)	C_15_H_16_N_4_O_6_	4.3	349.1150	349.1143	−2.12	305.1278	[36]
2	Asparagine-Bx (vulgaxanthin III)	C_13_H_15_N_3_O_7_	4.9	326.0990	326.0983	−2.23	-	[36]
3	Histamine-Bx	C_14_H_16_N_4_O_4_	5.4	305.1248	305.1244	−1.21	-	[37]
4	Glutamine-Bx (vulgaxanthin I)	C_14_H_17_N_3_O_7_	6.4	340.1145	340.1139	−1.69	323.0905	[38]
5	Ethanolamine-Bx	C_11_H_14_N_2_O_5_	7.5	255.0972	255.0975	1.37	-	[39]
6	Threonine-Bx	C_13_H_16_N_2_O_7_	8.5	313.1033	313.1030	−0.87	269.1061	[36]
7	Glutamic acid-Bx (vulgaxanthin II)	C_14_H_16_N_2_O_8_	9.0	341.0983	341.0979	−1.05	297.1100	[36]
8	Alanine-Bx	C_12_H_14_N_2_O_6_	11.4	283.0928	283.0925	−1.2	-	[37]
9	*γ*-Aminobutyric acid-Bx	C_13_H_16_N_2_O_6_	12.9	297.1085	297.1081	0.04	-	[39]
10	Proline-IBx (isoindicaxanthin)	C_14_H_16_N_2_O_6_	14.0	309.1083	309.1081	−0.61	265.1550	[40]
11	Proline-Bx (indicaxanthin)	C_14_H_16_N_2_O_6_	14.4	309.1085	309.1081	−1.26	265.1541	[40]
12	Dopa-Bx (dopaxanthin)	C_18_H_18_N_2_O_8_	15.9	391.1139	391.1136	−0.79	347.1568	[36]
13	Dopamine-Bx (miraxanthin V)	C_17_H_18_N_2_O_6_	18.0	347.1244	347.1238	−1.84	303.1230	[36]
14	Tyrosine-Bx (portulacaxanthin II)	C_18_H_18_N_2_O_7_	18.3	375.1179	375.1187	2.08	329.0998; 239.0896	[41]
15	Methionine-Bx	C_14_H_18_N_2_O_6_S	18.4	343.0966	343.0958	−2.24	299.1032	[36]
16	Valine-Bx	C_14_H_18_N_2_O_6_	19.6	311.1244	311.1238	−2.05	267.0984	[36]
17	Isoleucine-Bx	C_15_H_20_N_2_O_6_	21.9	325.1398	325.1394	−1.19	191.1427; 235.1437; 279.1353	[41]
18	Leucine-Bx (vulgaxanthin IV)	C_15_H_20_N_2_O_6_	22.1	325.1396	325.1394	−0.58	191.1547; 235.1490; 279.1412	[41]
19	Phenylalanine-Bx	C_18_H_18_N_2_O_6_	22.3	359.1243	359.1238	−1.5	313.1235; 223.1258; 131.1101	[41]
	***Betacyanins***							
20	Betanidin-5-*O*-*ß*-glucoside (Betanin)	C_24_H_26_N_2_O_13_	16.6	551.1510	551.1508	−0.43	389.1850	[40]
21	Isobetanidin-5-*O*-*ß*-glucoside (Isobetanin)	C_24_H_26_N_2_O_13_	17.4	551.1513	551.1508	−0.97	389.1880	[40]
				***m*/*z*** **[M-H]^−^_exp_**	***m*/*z*** **[M-H]^−^_caled_**		***m*/*z*** **from MS^2^ of [M-H]^−^**	
	***Organic acids and their derivatives***							
22	Malic acid	C_4_H_6_O_5_	1.7	133.0138	133.0142	3.33	115.0033	[42]
23	Gluconic acid	C_6_H_12_O_7_	1.8	195.0502	195.0510	4.21	177.0392; 129.0192	[42]
24	Citric acid	C_6_H_8_O_7_	2.4	191.0188	191.0197	4.82	111.0080	[43]
25	2-Hydroxyquinoline-3-carboxylic acid	C_10_H_7_NO_3_	9.5	188.0344	188.0353	4.85	170.9705; 144.0456	[42]
26	Benzoic acid	C_7_H_6_O_2_	13.5	121.0295	121.0295	0.02	109.0656	[44]
27	Feruloylmalic acid	C_14_H_14_O_8_	24.0	309.0612	309.0616	1.26	193.0507; 178.0272; 149.1213; 134.0366; 117.0305	[42]
28	Carnosic acid	C_20_H_28_O_4_	42.8	331.1915	331.1915	−0.05	290.9496; 259.2061; 236.9202; 201.0325	[44]
	***Amino acids and their derivatives***							
29	Tryptophan	C_11_H_12_N_2_O_2_	5.5	203.0830	203.0826	−1.95	142.0667; 116.0525	[45]
30	*N*-Benzoylaspartic acid	C_11_H_11_NO_5_	13.1	236.0565	236.0564	−0.23	192.0683; 174.0560; 148.0765; 120.0464	[42]
31	*N*-(Carboxyacetyl) phenylalanine	C_12_H_13_NO_5_	14.2	250.0722	250.0721	−0.41	206.0824;91.0552	[42]
	***Hydroxybenzoic acids and their derivatives***							
32	Dihydroxybenzoic acid	C_7_H_6_O_4_	7.4	153.0197	153.0193	0.21	109.0312	[44]
33	4-Hydroxybenzoic acid	C_7_H_6_O_3_	9.9	137.0248	137.0244	−2.77	109.0268	[44]
34	Vanillic acid hexoside	C_14_H_18_O_9_	15.4	329.0877	329.0878	0.32	167.0353; 123.0438	[44]
	***Hydroxycinnamic acids and their derivatives***							
35	Caffeic acid hexoside	C_15_H_18_O_9_	10.2	341.0882	341.0878	−1.15	179.0335; 161.0250	[46]
36	Ferulic acid hexose I	C_16_H_20_O_9_	12.2	355.1036	355.1035	−0.40	193.0508; 178.0362; 134.0358; 149.0584	[42]
37	Chlorogenic acid I	C_16_H_18_O_9_	13.4	353.0873	353.0878	1.54	191.0557; 179.0422; 173.0533; 135.0454	[45]
38	Ferulic acid hexose II	C_16_H_20_O_9_	13.7	355.1029	355.1035	1.56	193.0505; 178.0358; 134.0365; 149.0591	[42]
39	Chlorogenic acid II	C_16_H_18_O_9_	14.9	353.0885	353.0878	−1.96	191.0561; 179.0365; 173.0446; 135.0485	[45]
40	4-*p*-Coumaroylquinic acid I	C_16_H_18_O_8_	17.5	337.0920	377.0929	2.64	190.9935; 163.0414	[44]
41	Feruloylquinic acid	C_17_H_20_O_9_	18.9	367.1042	367.1035	−2.02	191.0554; 175.0499	[44]
42	1-*O*-Sinapoyl-beta-*D*-glucose	C_17_H_22_O_10_	19.1	385.1154	385.1140	−3.57	175.0403; 223.0148	[47]
43	4-*p*-Coumaroylquinic acid II	C_16_H_18_O_8_	19.2	337.0934	377.0929	−1.51	190.9928; 163.0407	[44]
44	*p*-Coumaric acid	C_9_H_8_O_3_	23.1	163.0399	163.0401	1.02	119.0478	[42]
45	Rosmarinic acid	C_18_H_16_O_8_	26.7	359.0772	359.0772	0.11	197.0424; 179.0445; 161.1013; 135.0527	[44]
	***Hydrolysable tannins and their derivatives***							
46	Galloyl hexoside	C_13_H_16_O_10_	11.8	331.0663	331.0671	2.32	169.0089; 125.0223	[48]
	***Fatty acid and their derivatives***							
47	Tuberonic acid hexoside	C_18_H_28_O_9_	13.7	387.1675	387.1661	−3.72	207.1041; 163.0432; 101.0227	[44]
48	Tuberonic acid	C_12_H_18_O_4_	16.0	225.1135	225.1132	−1.18	163.0460; 174.0846	[44]
49	Trihydroxyoctadecadienoic acid	C_18_H_32_O_5_	32.0	327.2173	327.2177	1.21	314.7395; 299.3326; 271.2080; 229.1379; 171.1009	[44]
50	Dihydroxyhexadecanoic acid	C_16_H_32_O_4_	41.4	287.2242	287.2228	−4.92	228.0024	[44]
51	Hydroperoxyoctadecadienoic acid	C_18_H_32_O_4_	44.7	311.2232	311.2228	−1.34	293.1788; 223.1338; 161.0425	[44]
52	Hydroxyoctadecatrienoic acid I	C_18_H_30_O_3_	46.0	293.2121	293.2122	0.40	275.2007; 183.1436; 171.0992; 121.1022	[44]
53	Hydroxyoctadecatrienoic acid II	C_18_H_30_O_3_	46.8	293.2118	293.2122	1.42	275.2007; 183.1435; 171.0992; 121.1021	[44]
54	Hydroxyoctadecatrienoic acid III	C_18_H_30_O_3_	47.1	293.2128	293.2122	−1.98	275.1915; 183.1427; 171.0987; 121.1018	[44]
55	Hydroxyoctadecadienoic acid I	C_18_H_32_O_3_	49.4	295.2279	295.2279	−0.11	277.2162; 181.0419; 171.9458	[44]
56	Hydroxyoctadecadienoic acid II	C_18_H_32_O_3_	49.5	295.2273	295.2279	1.92	277.2162; 181.0410; 171.9480	[44]
	***Flavones and their derivatives***							
57	Luteolin-6,8-*C*-dihexose	C_27_H_30_O_16_	20.6	609.1462	609.1461	−0.15	285.0370; 255.1022	[49]
	***Flavonoid and their derivatives***							
58	Quercetin-*O*-hexoside I	C_21_H_20_O_12_	23.0	463.0886	463.0882	−0.86	301.0333	[43]
59	Luteolin-7-*O*-rutinoside I	C_27_H_30_O_15_	23.4	593.1521	593.1512	−1.53	285.0356	[46]
60	Quercetin-*O*-hexoside II	C_21_H_20_O_12_	23.9	463.0877	463.0882	1.08	301.0324	[43]
61	Luteolin-7-*O*-rutinoside II	C_27_H_30_O_15_	24.2	593.1520	593.1512	−1.36	285.0391	[50]
62	Luteolin-7-*O*-rutinoside III	C_27_H_30_O_15_	24.6	593.1523	593.0561	−1.86	285.0375	[46]
63	Luteolin-*O*-hexoside	C_21_H_20_O_11_	25.4	447.0934	447.0933	−0.26	285.0373	[44]
64	Genistein-4′-*O*-glucoside	C_21_H_20_O_10_	25.8	431.0980	431.0984	0.86	431.0980; 269.1377	[42]
65	Apigenin I	C_15_H_10_O_5_	28.9	269.0461	269.0455	−2.05	151.1127; 127.0782	[44]
66	Luteolin	C_15_H_10_O_6_	31.0	285.0404	285.0405	0.22	267.0259; 257.0436; 243.0286; 241.0500; 217.0480; 199.0380; 197.0569; 175.0380; 151.0016; 133.0285	[48]
67	Naringenin	C_15_H_12_O_5_	33.5	271.0612	271.0612	−0.01	227.2072; 177.0298; 151.0005; 119.0365	[44]
68	Apigenin II	C_15_H_10_O_5_	34.0	269.0455	269.0455	0.17	151.1149; 127.0725	[44]
69	Cirsimaritin	C_17_H_14_O_6_	34.1	313.0707	313.0718	3.38	298.9546; 283.0735; 255.0615	[44]
70	Sorbifolin	C_16_H_12_O_6_	34.2	299.0557	299.0561	1.37	284.0365; 271.0601	[44]
71	Kaempferol	C_15_H_10_O_6_	34.4	285.0407	285.0405	−0.83	257.0438; 151.0030	[48]

**Table 3:** Antioxidant properties of yellow, orange, red and purple flowers of *P. grandiflora* determined by ABTS, FRAP and DPPH assays and correlation coefficients between identified metabolites (absolute peak areas) and antioxidant activity (mM TE/g DE). Statistical significance is marked by font: boldface means 95% significance and very strong correlation (R = 0.7–1.0); italic font means 95% significance and strong correlation (R= 0.5–0.7).

Antioxidant Activity	ABTS	FRAP	DPPH
	(mM TE/g DE) *		
Yellow	0.079 ± 0.015 ^a^	0.066 ± 0.010 ^a^	0.078 ± 0.016 ^a^
Orange	0.112 ± 0.017 ^b^	0.067 ± 0.012 ^a^	0.111 ± 0.064 ^b^
Red	0.176 ± 0.020 ^d^	0.160 ± 0.005 ^b^	0.174 ± 0.057 ^d^
Purple	0.135 ± 0.016 ^c^	0.077 ± 0.004 ^c^	0.125 ± 0.035 ^c^
Ascorbic acid	7.19 ± 0.025 ^c^	3.36 ± 0.005 ^c^	8.07 ± 0.035 ^c^
Fisher’ LSD	0.001	0.002	0.002
**Correlation**			
***Betaxanthins***			
Histidine-Bx	−0.495	−0.020	−0.440
Asparagine-Bx	−0.035	−0.466	−0.136
Histamine-Bx	−0.546	−0.884	−0.606
Glutamine-Bx	−0.214	−0.056	−0.124
Ethanolamine-Bx	−0.562	−0.840	−0.585
Threonine-Bx	−0.579	−0.792	−0.581
Glutamic acid-Bx	−0.584	−0.635	−0.545
Alanine-Bx	−0.852	−0.920	−0.848
*γ*-Aminobutyric acid-Bx	−0.482	−0.631	−0.458
Proline-IBx	−0.295	−0.416	−0.254
Proline-Bx	−0.726	−0.736	−0.691
Dopa-Bx	−0.275	−0.688	−0.365
Dopamine-Bx	0.040	−0.443	−0.049
Tyrosine-Bx	−0.844	−0.865	−0.893
Methionine-Bx	−0.869	−0.615	−0.812
Valine-Bx	−0.591	−0.822	−0.600
Isoleucine-Bx	−0.606	−0.750	−0.591
Leucine-Bx	−0.687	−0.759	−0.662
Phenylalanine-Bx	−0.916	−0.917	−0.906
***Betacyanins***			
Betanidin-5-*O*-*ß*-glucoside	0.273	−0.122	0.170
Isobetanidin-5-*O*-*ß*-glucoside	−0.296	−0.536	−0.435
***Organic acids and their derivatives***			
Malic acid	**0.878**	*0.652*	**0.824**
Gluconic acid	−0.315	0.102	−0.292
Citric acid	−0.628	−0.422	−0.551
2-Hydroxyquinoline-3-carboxylic acid	0.114	−0.245	0.009
Benzoic acid	0.157	−0.229	0.052
Feruloylmalic acid	0.078	−0.308	0.063
Carnosic acid	−0.182	−0.032	−0.229
***Amino acids and their derivatives***			
Tryptophan	−0.120	−0.161	−0.199
*N*-Benzoylaspartic acid	−0.030	0.428	0.068
*N*-(Carboxyacetyl) phenylalanine	**0.977**	**0.958**	**0.993**
***Hydroxybenzoic acids and their derivatives***			
Dihydroxybenzoic acid	−0.821	−0.746	−0.862
Hydroxybenzoic acid	*0.530*	0.098	0.440
Vanillic acid hexoside	−0.421	0.048	−0.330
***Hydroxycinnamic acids and their derivatives***			
Caffeic acid hexoside	−0.874	−0.597	−0.868
Ferulic acid hexose I	−0.291	0.076	−0.188
Chlorogenic acid I	−0.508	−0.103	−0.415
Ferulic acid hexose II	−0.583	−0.141	−0.500
Chlorogenic acid II	−0.214	−0.606	−0.312
4-*p*-Coumaroylquinic acid I	−1.000	−0.968	−0.990
Feruloylquinic acid	**0.806**	**0.754**	**0.767**
1-*O*-Sinapoyl-beta-*D*-glucose	−0.127	−0.307	−0.093
4-*p*-Coumaroylquinic acid II	−0.061	−0.480	−0.094
*p*-Coumaric acid	−0.225	−0.276	−0.167
Rosmarinic acid	**0.756**	**0.978**	**0.799**
***Hydrolysable tannins and their derivatives***			
Galloyl hexoside	**0.775**	**0.919**	**0.838**
***Fatty acids and their derivatives***			
Tuberonic acid hexoside	*0.531*	0.160	*0.521*
Tuberonic acid	0.172	−0.156	0.068
Trihydroxyoctadecadienoic acid	**0.731**	**0.845**	**0.722**
Dihydroxyhexadecanoic acid	*0.642*	0.396	*0.563*
Hydroperoxyoctadecadienoic acid	**0.769**	**0.841**	**0.753**
Hydroxyoctadecatrienoic acid I	**0.919**	**0.828**	**0.890**
Hydroxyoctadecatrienoic acid II	0.469	0.471	0.414
Hydroxyoctadecatrienoic acid III	0.496	0.298	0.413
Hydroxyoctadecadienoic acid I	0.349	0.065	0.251
Hydroxyoctadecadienoic acid II	0.193	*0.622*	0.286
***Flavones and their derivatives***			
Luteolin-6,8-*C*-dihexose	0.371	0.325	0.436
***Flavonoids and their derivatives***			
Quercetin-*O*-hexoside I	**0.836**	**0.995**	**0.879**
Luteolin-7-*O*-rutinoside I	−0.837	−0.465	−0.789
Quercetin-*O*-hexoside II	**0.912**	*0.650*	**0.904**
Luteolin-7-*O*-rutinoside II	*0.522*	*0.659*	*0.605*
Luteolin-7-*O*-rutinoside III	−0.919	−0.932	−0.913
Luteolin-*O*-hexoside	−0.350	−0.242	−0.405
Genistein-4′-*O*-glucoside	0.252	0.353	0.339
Apigenin I	0.055	−0.138	0.092
Luteolin	**0.803**	**0.745**	**0.763**
Naringenin	0.415	**0.783**	*0.500*
Apigenin II	0.419	**0.754**	*0.510*
Cirsimaritin	**0.935**	**0.810**	**0.903**
Sorbifolin	*0.591*	0.850	*0.610*
Kaempferol	0.327	0.452	0.288

* DE = Dry extract; TE = Trolox equivalents. Each value represents the mean and ± standard. deviation from three lots. Values in each column having the same letter are not significantly different at *p* < 0.05.

**Table 4:** Antimicrobial activity of extracts from the yellow, orange, red and purple flowers of *Portulaca grandiflora* assessed for their MIC (minimum inhibitory concentration), MBC (minimum bactericidal concentration), MFC (minimum fungicidal concentration) and correlation coefficients between identified metabolites (absolute peak areas) and microbial activity (MIC values).

	*Gram-Positive Bacteria*								*Gram-Negative Bacteria*			*Fungal (Yeasts) Strains*		
*Microorganism/* *Metabolite*	*S. aureus * *ATCC 29213*	*S. aureus * *ATCC 25923*	*S. aureus * *ATCC 6538*	*S. aureus * *ATCC BAA-1707*	*S. epidermidis * *ATCC 12228*	*M. luteus * *ATCC 10240*	*B. subtilis* *ATCC 6633*	*B. cereus* *ATCC 10876*	*E. coli* *ATCC 25922*	*S. typhimurium* *ATCC 14028*	*P. aeruginosa* *ATCC 27853*	*C. albicans* *ATCC 10231*	*C. glabrata* *ATCC 90030*	*C. krusei* *ATCC 14243*
	(MIC;MBC)								(MIC;MBC)			(MIC;MFC)		
Yellow	8;32	8;32	8;32	8;32	8;32	4;16	4;16	8;16	16;16	16;16	16;16	32;32	32;32	32;32
Orange	8;32	8;32	8;32	8;32	8;32	4;16	4;16	8;16	16;32	16;16	16;16	32;32	32;32	32;32
Red	16;32	32;32	32;32	32;32	32;32	16;16	16;16	16;16	16;32	16;16	16;16	32;32	32;32	32;32
Purple	>32;>32	32;32	32;32	>32;>32	>32;>32	32;32	16;16	32;32	16;32	16;32	16;32	32;32	32;32	32;32
**Correlation**														
***Betaxanthins***														
Histidine-Bx	**−0.609**	**−0.467**	**−0.467**	**−0.466**	**−0.466**	**−0.495**	**−0.233**	**−0.609**	**−0.079**	**−0.478**	**−0.940**	**−0.651**	**−0.534**	**−0.885**
Asparagine-Bx	0.811	0.339	0.340	0.339	0.339	0.750	0.280	0.811	0.016	0.163	0.679	0.806	0.760	0.933
Histamine-Bx	0.232	**−0.340**	**−0.340**	**−0.341**	**−0.341**	0.138	**−0.415**	0.232	0.653	0.644	0.682	0.228	0.993	0.656
Glutamine-Bx	**−0.878**	**−0.696**	**−0.696**	**−0.696**	**−0.696**	**−0.930**	**−0.800**	**−0.878**	0.689	0.643	0.017	**−0.849**	**−0.043**	**−0.501**
Ethanolamine-Bx	**−0.103**	**−0.568**	**−0.568**	**−0.568**	**−0.568**	**−0.218**	**−0.693**	**−0.103**	0.886	0.882	0.692	**−0.092**	0.933	0.457
Threonine-Bx	**−0.288**	**−0.679**	**−0.679**	**−0.680**	**−0.680**	**−0.401**	**−0.809**	**−0.288**	0.957	0.933	0.619	**−0.273**	0.842	0.299
Glutamic acid-Bx	**−0.623**	**−0.840**	**−0.840**	**−0.841**	**−0.841**	**−0.718**	**−0.953**	**−0.623**	0.990	0.909	0.378	**−0.605**	0.573	**−0.059**
Alanine-Bx	**−0.425**	**−0.855**	**−0.855**	**−0.855**	**−0.855**	**−0.496**	**−0.880**	**−0.425**	0.937	0.740	0.298	**−0.433**	0.751	0.057
*γ*-Aminobutyric acid-Bx	**−0.467**	**−0.721**	**−0.721**	**−0.722**	**−0.722**	**−0.581**	**−0.874**	**−0.467**	0.976	0.972	0.557	**−0.442**	0.679	0.133
Proline-IBx	**−0.534**	**−0.648**	**−0.648**	**−0.648**	**−0.648**	**−0.649**	**−0.831**	**−0.534**	0.910	0.966	0.538	**−0.499**	0.512	0.050
Proline-Bx	**−0.640**	**−0.911**	**−0.911**	**−0.912**	**−0.912**	**−0.719**	**−0.978**	**−0.641**	0.990	0.833	0.269	**−0.634**	0.586	**−0.115**
Dopa-Bx	0.606	0.055	0.055	0.055	0.055	0.528	−0.010	0.605	0.297	0.378	0.712	0.600	0.911	0.864
Dopamine-Bx	0.721	0.284	0.284	0.283	0.283	0.626	0.150	0.721	0.177	0.400	0.862	0.733	0.829	0.979
Tyrosine-Bx	0.080	**−0.440**	**−0.440**	**−0.440**	**−0.440**	0.095	−0.293	0.080	0.325	0.022	**−0.067**	0.033	0.578	0.142
Methionine-Bx	**−0.835**	**−0.986**	**−0.986**	**−0.985**	**−0.985**	**−0.834**	**−0.904**	**−0.836**	0.743	0.391	**−0.309**	**−0.855**	0.183	**−0.574**
Valine-Bx	**−0.229**	**−0.652**	**−0.652**	**−0.653**	**−0.653**	**−0.342**	**−0.776**	**−0.229**	0.938	0.913	0.636	**−0.216**	0.878	0.348
Isoleucine-Bx	**−0.439**	**−0.769**	**−0.769**	**−0.769**	**−0.769**	**−0.545**	**−0.890**	**−0.439**	0.991	0.935	0.518	**−0.424**	0.745	0.146
Leucine-Bx	**−0.549**	**−0.856**	**−0.856**	**−0.856**	**−0.857**	**−0.640**	**−0.945**	**−0.549**	0.999	0.883	0.384	**−0.539**	0.671	0.007
Phenylalanine-Bx	**−0.487**	**−0.900**	**−0.900**	**−0.900**	**−0.900**	**−0.539**	**−0.886**	**−0.487**	0.900	0.646	0.156	**−0.502**	0.670	**−0.060**
***Betacyanins***														
Betanidin-5-*O*-*ß*-glucoside	0.971	0.660	0.661	0.660	0.660	0.941	0.619	0.971	**−0.350**	**−0.140**	0.540	0.968	0.465	0.897
Isobetanidin-5-*O*-*ß*-glucoside	0.890	0.375	0.375	0.374	0.374	0.836	0.375	0.890	**−0.141**	**−0.141**	0.549	0.890	0.616	0.971
***Organic acids and their derivatives***														
Malic acid	0.817	0.993	0.993	0.993	0.993	0.824	0.924	0.817	**−0.782**	**−0.442**	0.249	0.835	−0.241	0.524
Gluconic acid	**−0.242**	**−0.119**	**−0.119**	**−0.118**	**−0.118**	**−0.106**	0.148	−0.242	**−0.419**	**−0.771**	**−0.955**	**−0.292**	**−0.589**	**−0.695**
Citric acid	**−0.926**	**−0.944**	**−0.944**	**−0.944**	**−0.944**	**−0.960**	**−0.960**	**−0.926**	0.825	0.605	**−0.121**	**−0.921**	0.136	**−0.551**
2-Hydroxyquinoline-3-carboxylic acid	0.934	0.562	0.562	0.562	0.562	0.916	0.561	0.934	**−0.313**	**−0.179**	0.452	0.921	0.501	0.845
Benzoic acid	0.942	0.577	0.577	0.576	0.576	0.914	0.552	0.942	**−0.288**	**−0.121**	0.521	0.934	0.527	0.885
Feruloylmalic acid	0.082	**−0.122**	**−0.122**	**−0.123**	**−0.123**	**−0.061**	**−0.375**	0.082	0.629	0.900	0.936	0.127	0.707	0.621
Carnosic acid	0.388	0.278	0.278	0.278	0.278	0.502	0.518	0.388	**−0.608**	**−0.833**	**−0.584**	**0.334**	**−0.238**	**−0.093**
***Amino acids and their derivatives***														
Tryptophan	0.666	0.411	0.411	0.411	0.411	0.740	0.590	0.665	−0.550	−0.669	**−0.235**	**0.619**	0.060	0.286
*N*-Benzoylaspartic acid	**−0.805**	**−0.356**	**−0.357**	**−0.356**	**−0.356**	**−0.731**	**−0.266**	**−0.805**	**−0.047**	**−0.237**	**−0.757**	**−0.807**	**−0.773**	**−0.967**
*N*-(Carboxyacetyl)-phenylalanine	0.204	0.795	0.795	0.795	0.796	0.204	0.795	0.205	**−0.992**	**−0.511**	0.205	0.250	−0.606	0.063
***Hydroxybenzoic acids and their derivatives***														
Dihydroxybenzoic acid	0.035	**−0.404**	**−0.404**	**−0.404**	**−0.404**	0.082	−0.208	0.034	0.176	−0.176	**−0.285**	**−0.020**	0.377	**−0.025**
Hydroxybenzoic acid	0.950	0.771	0.771	0.770	0.770	0.898	0.656	0.950	**−0.369**	**−0.039**	0.667	0.965	0.375	0.922
Vanillic acid hexoside	**−0.902**	**−0.649**	**−0.649**	**−0.649**	**−0.649**	**−0.831**	**−0.514**	**−0.902**	0.201	**−0.122**	**−0.773**	**−0.919**	**−0.527**	**−0.975**
***Hydroxycinnamic acids and their derivatives***														
Caffeic acid hexoside	**−0.378**	**−0.628**	**−0.628**	**−0.627**	**−0.627**	**−0.305**	**−0.401**	**−0.378**	0.231	**−0.210**	**−0.594**	**−0.431**	0.071	**−0.465**
Ferulic acid hexose I	**−0.983**	**−0.696**	**−0.696**	**−0.696**	**−0.696**	**−0.965**	**−0.674**	**−0.983**	0.424	0.228	**−0.465**	**−0.976**	**−0.396**	**−0.855**
Chlorogenic acid I	**−0.980**	**−0.797**	**−0.797**	**−0.796**	**−0.796**	**−0.943**	**−0.712**	**−0.980**	0.441	0.141	**−0.585**	**−0.989**	**−0.332**	**−0.892**
Ferulic acid hexose II	**−0.910**	**−0.765**	**−0.765**	**−0.765**	**−0.764**	**−0.848**	**−0.623**	**−0.910**	0.330	**−0.032**	**−0.719**	**−0.932**	**−0.367**	**−0.921**
Chlorogenic acid II	0.720	0.190	0.190	0.189	0.189	0.664	0.160	0.720	0.113	0.184	0.610	0.707	0.812	0.861
4-*p*-Coumaroylquinic acid I	**−0.162**	**−0.768**	**−0.768**	**−0.768**	**−0.769**	**−0.267**	**−0.768**	**−0.162**	0.900	0.900	0.964	**−0.162**	0.938	0.523
Feruloylquinic acid	0.693	0.960	0.960	0.961	0.961	0.753	0.986	0.693	**−0.959**	**−0.740**	**−0.132**	0.694	**−0.522**	0.220
1-*O*-Sinapoyl-beta-*D*-glucose	**−0.417**	**−0.497**	**−0.497**	**−0.497**	**−0.497**	**−0.542**	**−0.715**	**−0.417**	0.830	0.967	0.643	**−0.374**	0.503	0.161
4-*p*-Coumaroylquinic acid II	0.182	**−0.145**	**−0.145**	**−0.146**	**−0.146**	0.042	−0.370	0.182	0.650	0.877	0.957	0.217	0.847	0.719
*p*-Coumaric acid	**−0.640**	**−0.646**	**−0.646**	**−0.646**	**−0.646**	**−0.742**	**−0.823**	**−0.640**	0.852	0.904	0.421	**−0.602**	0.343	−0.100
Rosmarinic acid	**−0.008**	0.563	0.563	0.563	0.563	0.067	0.587	−0.008	**−0.749**	**−0.621**	**−0.480**	0.006	**−0.931**	**−0.424**
***Hydrolysable tannins and their derivatives***														
Galloyl hexoside	**−0.199**	0.382	0.382	0.382	0.382	−0.181	0.296	−0.199	**−0.410**	**−0.194**	**−0.188**	**−0.163**	**−0.759**	**−0.365**
***Fatty acids and their derivatives***														
Tuberonic acid hexoside	0.237	0.267	0.266	0.266	0.266	0.114	−0.007	0.237	0.244	0.644	0.865	0.293	0.359	0.601
Tuberonic acid	0.954	0.629	0.629	0.629	0.629	0.950	0.645	0.954	**−0.419**	**−0.288**	0.370	0.938	0.399	0.796
Trihydroxyoctadecadienoic acid	0.434	0.820	0.820	0.820	0.820	0.526	0.898	0.434	**−0.983**	**−0.861**	**−0.429**	0.429	**−0.766**	**−0.114**
Dihydroxyhexadecenoic acid	0.388	0.278	0.278	0.278	0.278	0.502	0.518	0.388	**−0.608**	**−0.833**	−0.584	0.334	**−0.238**	**−0.093**
Hydroperoxyoctadecadienoic acid	0.949	0.946	0.946	0.946	0.946	0.970	0.939	0.949	**−0.778**	**−0.530**	0.210	0.948	**−0.070**	0.619
Hydroxyoctadecatrienoic acid I	0.497	0.865	0.865	0.865	0.865	0.581	0.927	0.497	**−0.984**	**−0.831**	**−0.353**	0.495	**−0.718**	−0.033
Hydroxyoctadecatrienoic acid II	0.647	0.968	0.968	0.968	0.968	0.684	0.936	0.647	**−0.886**	**−0.594**	**−0.002**	0.662	**−0.512**	0.255
Hydroxyoctadecatrienoic acid III	0.717	0.816	0.816	0.817	0.817	0.807	0.941	0.717	**−0.942**	**−0.885**	**−0.312**	0.693	**−0.415**	0.177
HydroxyOctadecadienoic acid I	0.936	0.879	0.879	0.879	0.879	0.975	0.925	0.936	**−0.792**	**−0.627**	0.084	0.922	**−0.068**	0.555
Hydroxyoctadecadienoic acid II	0.980	0.776	0.777	0.776	0.776	0.993	0.801	0.980	**−0.607**	**−0.444**	0.265	0.966	0.193	0.718
***Flavones and their derivatives***														
Luteolin−6.8-*C*-dihexose	**−0.445**	**−0.150**	**−0.150**	**−0.151**	**−0.151**	**−0.528**	**−0.368**	**−0.445**	0.384	0.618	0.370	**−0.390**	**−0.082**	**−0.105**
***Flavonoids and their derivatives***														
Quercetin-*O*-hexoside I	0.008	0.584	0.583	0.584	0.584	0.059	0.556	0.008	**−0.678**	−0.482	**−0.322**	0.032	**−0.859**	**−0.329**
Luteolin-7-*O*-rutinoside I	**−0.715**	**−0.806**	**−0.806**	**−0.806**	**−0.806**	**−0.652**	**−0.616**	**−0.716**	0.378	**−0.059**	**−0.661**	**−0.756**	**−0.091**	**−0.723**
Quercetin-*O*-hexoside II	0.413	0.685	0.685	0.685	0.685	0.351	0.472	0.413	**−0.313**	0.126	0.541	0.464	**−0.132**	0.448
Luteolin-7-*O*-rutinoside II	**−0.501**	0.003	0.002	0.002	0.002	**−0.523**	**−0.132**	**−0.501**	0.024	0.199	**−0.046**	**−0.457**	**−0.535**	**−0.423**
Luteolin-7-*O*-rutinoside III	**−0.455**	**−0.884**	**−0.884**	**−0.884**	**−0.884**	**−0.507**	**−0.869**	**−0.455**	0.890	0.638	0.168	**−0.471**	0.690	**−0.032**
Luteolin-*O*-hexoside	0.373	0.145	0.145	0.145	0.145	0.471	0.382	0.373	**−0.441**	**−0.697**	**−0.491**	0.316	**−0.046**	**−0.018**
Genistein-4’-*O*-glucoside	**−0.660**	**−0.295**	**−0.296**	**−0.296**	**−0.296**	**−0.713**	**−0.454**	**−0.660**	0.373	0.500	0.097	**−0.615**	**−0.265**	**−0.391**
Apigenin I	**−0.356**	**−0.353**	**−0.353**	**−0.353**	**−0.353**	**−0.481**	**−0.593**	**−0.356**	0.714	0.917	0.664	**−0.306**	0.402	0.183
Luteolin	0.703	0.963	0.963	0.963	0.963	0.762	0.988	0.703	**−0.957**	**−0.737**	**−0.123**	0.704	**−0.510**	0.233
Naringenin	**−0.514**	0.068	0.068	0.068	0.069	**−0.444**	0.105	**−0.514**	**−0.363**	**−0.377**	**−0.629**	**−0.502**	**−0.924**	**−0.778**
Apigenin II	**−0.571**	0.008	0.008	0.008	0.008	**−0.521**	0.007	**−0.570**	**−0.244**	**−0.226**	**−0.519**	**−0.550**	**−0.853**	**−0.745**
Cirsimaritin	0.669	0.974	0.974	0.974	0.974	0.695	0.923	0.669	**−0.851**	**−0.535**	0.073	0.688	**−0.458**	0.313
Sorbifolin	0.143	0.604	0.604	0.604	0.604	0.255	0.722	0.143	**−0.903**	**−0.883**	**−0.662**	0.133	**−0.920**	**−0.417**
Kaempferol	0.527	0.663	0.663	0.663	0.664	0.642	0.842	0.527	**−0.924**	**−0.971**	**−0.542**	0.493	**−0.540**	**−0.062**

MIC, MBC and MFC were expressed as mg/mL. The representative data (mode) are present. The positive impact on antibacterial activity is marked in boldface.

**Table 5:** The reduction of HHV-1 infectious titer and viral load by *P. grandiflora* extracts.

Sample	Variety	Concentration (µg/mL)	Decrease of HHV-1 Infectious Titer (Δlog) *		Decrease of HHV-1 Viral Load (Δlog’) *
*P. grandiflora*	yellow	62	0.69	±0.22	0.44
	orange		0.45	±0.25	0.32
	red		0.5	±0.14	0.17
	purple		0.2	±0.07	0.22
Acyclovir	n/a	60	>3.5		> 7

* Δlog (mean ± SD)—mean was calculated from titration assays of samples collected from two independent antiviral assays. Δlog = logCCID_50_VC − logCCID_50_TE; VC—virus control; TE—tested extract, Δlog of at least three is regarded significant. Δlog’ = logViralLoadVC−logViralLoadTE; VC—virus control; TE—tested extract, Viral Load—copies/µL n/a—not applicable.

## Data Availability

The data presented in this study are available on request from the corresponding authors.

## References

[1] Shinde P.R., Wagh K.R., Patil P.S., Bairagi V.A. (2014). Pharmacognostic standardization and antibacterial potential of aerial herbs of *Portulaca grandiflora* Hooker (Portulaceae). World J. Pharm. Sci..

[2] Ohsaki A., Asaka Y., Kubota T., Shibata K., Tokoroyama T. (1997). Portulene acetal, a novel minor constituent of *Portulaca grandiflora* with significance for the biosynthesis of portulal. J. Nat. Prod..

[3] Chavalittumrong P., Sriwanthana B., Rojanawiwat A., Kijphati R., Jitjuk B., Treesangsri W., Phadungpat S., Bansiddhi J., Bunjob M. (2007). Safety of the aqueous extract of *Portulaca grandiflora* Hook. in healthy volunteers. Songklanakarin. J. Sci. Technol..

[4] Jayamohan N., Pawan Kumar P., Jayachandra K. (2013). Surveillance of *in vitro* antioxidant and anthelmintic activity of methanolic extract of *Syzygium cumini* Bark (Myrtaceae). Int. J. Phytopharm..

[5] Anghel A.I., Olaru O.T., Gatea F., Dinu M., Ancuceanu R.V., Istudor V. (2013). Preliminary research on *Portulaca grandiflora* Hook. species (Portulacaceae) for therapeutic use. Farmacia.

[6] Felippe M.J.B. (2014). Immunotherapy. Equine Infectious Diseases.

[7] Subramani P.A., Michael R.D. (2017). Prophylactic and prevention methods against diseases in aquaculture. Fish Diseases.

[8] Anderson D., Bishop J.B., Garner R.C., Ostrosky-Wegman P., Selby P.B. (1995). Cyclophosphamide: Review of its mutagenicity for an assessment of potential germ cell risks. Mutat. Res. Fundam. Mol. Mech. Mutagen..

[9] Spórna-Kucab A., Wróbel N., Kumorkiewicz-Jamro A., Wybraniec S. (2020). Separation of betacyanins from *Iresine herbstii* Hook. ex Lindl. leaves by high-speed countercurrent chromatography in a polar solvent system. J. Chromatogr. A.

[10] Spórna-Kucab A., Kumorkiewicz A., Szmyr N., Szneler E., Wybraniec S., Wybraniec S. (2019). Separation of betacyanins from flowers of *Amaranthus cruentus* L. in a polar solvent system by high-speed counter-current chromatography. J. Sep. Sci..

[11] Spórna-Kucab A., Milo A., Kumorkiewicz A., Wybraniec S. (2018). Studies on polar high-speed counter-current chromatographic systems in separation of amaranthine-type betacyanins from *Celosia* species. J. Chromatogr. B.

[12] Spórna-Kucab A., Jagodzińska J., Sławomir W. (2017). Separation of betacyanins from purple flowers of *Gomphrena globosa* L. by ion-pair high-speed counter-current chromatography. J. Chromatogr. A.

[13] Spórna-Kucab A., Hołda E., Wybraniec S. (2016). High-speed counter-current chromatography in separation of betacyanins from flowers of red *Gomphrena globosa* L. cultivars. J. Chromatogr. B.

[14] Böhm H., Böhm L., Rink E. (1991). Establishment and characterization of a betaxanthin-producing cell culture from *Portulaca grandiflora*. Plant Cell. Tissue Organ Cult..

[15] Trezzini G.F., Zrÿd J.P. (1991). Two betalains from *Portulaca grandiflora*. Phytochemistry..

[16] Madadi E., Mazloum-Ravasan S., Yu J.S., Ha J.W., Hamishehkar H., Kim K.H. (2020). Therapeutic application of betalains: A review. Plants.

[17] Erkan N. (2012). Antioxidant activity and phenolic compounds of fractions from *Portulaca oleracea* L.. Food Chem..

[18] Cai Y., Sun M., Corke H. (2003). Antioxidant activity of betalains from plants of the Amaranthaceae. J. Agric. Food Chem..

[19] Wybraniec S., Stalica P., Spórna-Kucab A., Nemzer B., Pietrzkowski Z., Michałowski T. (2011). Antioxidant activity of betanidin: Electrochemical study in aqueous media. J. Agric. Food Chem..

[20] Osorio-Esquivel O., Ortiz-Moreno A., Álvarez V.B., Dorantes-Álvarez L., Giusti M.M. (2011). Phenolics, betacyanins and antioxidant activity in *Opuntia joconostle* fruits. Food Res. Int..

[21] Khan M.I. (2016). Plant betalains: Safety, antioxidant activity, clinical efficacy, and bioavailability. Compr. Rev. Food Sci. Food Saf..

[22] Rahimi P., Abedimanesh S., Mesbah-Namin S.A., Ostadrahimi A. (2019). Betalains, the nature-inspired pigments, in health and diseases. Crit. Rev. Food Sci. Nutr..

[23] Pietrzkowski Z., Nemzer B., Spórna A., Stalica P. (2010). Influence of betalain-rich extract on reduction of discomfort associated with osteoarthritis. New Med..

[24] Nemzer B., Pietrzkowski Z., Spórna A., Stalica P. (2011). Betalainic and nutritional profiles of pigment-enriched red beet root (*Beta vulgaris* L.) dried extracts. Food Chem..

[25] Spórna-Kucab A., Bernaś K., Grzegorczyk A., Malm A., Skalicka-Woźniak K. (2018). Wybraniec, Liquid chromatographic techniques in betacyanin isomers separation from *Gomphrena globosa* L. flowers for the determination of their antimicrobial activities. S. J. Pharm. Biomed. Anal..

[26] Gómez-García M., Sol C., de Nova P.J., Puyalto M., Mesas L., Puente H., Mencía-Ares O., Miranda R., Argüello H., Rubio P. (2019). Antimicrobial activity of a selection of organic acids, their salts and essential oils against swine enteropathogenic bacteria. Porc. Health Manag..

[27] Wybraniec S., Stalica P., Jerz G., Klose G., Gebers N., Winterhalter P., Mizrahi Y. (2009). Separation of polar betalain pigments from cacti fruits of *Hylocereus polyrhizus* by ion-pair high-speed countercurrent chromatography. J. Chromatogr. A.

[28] Otálora C.M., Bonifazi E.L., Fissore E.N., Basanta M.F., Gerschenson L.N. (2020). Thermal stability of betalains in by-products of the blanching and cutting of *Beta vulgaris* L. var conditiva. Polish J. Food Nutr. Sci..

[29] Nilsson T. (1970). Studies into the pigments in beetroot (*Beta vulgaris* L. ssp. vulgaris var. rubra L.). Lantbr. Ann..

[30] Cai Y., Sun M., Schliemann W., Corke H. (2001). Chemical stability and colorant properties of betaxanthin pigments from *Celosia* argentea. J. Agric. Food Chem..

[31] Emad A.M., Rasheed D.M., El-Kased R.F., El-Kersh D.M. (2022). Antioxidant, antimicrobial activities and characterization of polyphenol-enriched extract of egyptian celery (*Apium graveolens* L., Apiaceae) aerial parts *via* UPLC. Molecules.

[32] Petkova R., Tcholakova S., Denkov N.D. (2012). Foaming and foam stability for mixed polymer-surfactant solutions: Effects of surfactant type and polymer charge. Langmuir.

[33] Dudonné S., Vitrac X., Coutiére P., Woillez M., Mérillon J.M. (2009). Comparative study of antioxidant properties and total phenolic content of 30 plant extracts of industrial interest using DPPH, ABTS, FRAP, SOD, and ORAC assays. J. Agric. Food Chem..

[34] EUCAST (2003). European committee for antimicrobial susceptibility testing (EUCAST) of the european society of clinical microbiology and infectious diseases (ESCMID): Determination of minimum inhibitory concentrations (MICs) of antibacterial agents by broth dilution. Clin. Microbiol. Infect. Dis..

[35] Malm A., Grzegorczyk A., Biernasiuk A., Baj T., Rój E., Tyśkiewicz K., Dębczak A., Stolarski M., Krzyżaniak J.M., Olba-Zięty E. (2020). Could supercritical extracts from the aerial parts of *Helianthus salicifolius* a. Dietr. and *Helianthus tuberosus* L. be regarded as potential raw materials for biocidal purposes. Agriculture.

[36] Svenson J., Smallfield B.M., Joyce N.I., Sansom C.E., Perry N.B. (2008). Betalains in red and yellow varieties of the andean tuber crop ulluco (*Ullucus tuberosus*). J. Agric. Food Chem..

[37] Kugler F., Stintzing F.C., Carle R. (2004). Identification of betalains from petioles of differently colored swiss chard (*Beta vulgaris* L. ssp. cicla Alef. Cv. Bright Lights) by high-performance liquid. J. Agric. Food Chem..

[38] Slatnar A., Stampar F., Veberic R., Jakopic J. (2015). HPLC-MSn Identification of betalain profile of different beetroot (*Beta vulgaris* L. ssp. vulgaris) parts and cultivars. J. Food Sci..

[39] Ho Suh D., Lee S., Yeon Heo D., Kim Y.-S., Kim Cho S., Lee S., Hwan Lee C. (2014). Metabolite profiling of red and white pitayas (*Hylocereus polyrhizus* and *Hylocereus undatus*) for comparing betalain biosynthesis and antioxidant activity. J. Agric. Food Chem..

[40] Barkociová M., Tóth J., Sutor K., Drobnicka N., Wybraniec S., Dudík B., Bilková A., Czigle S., Braca A., De Leo M. (2021). Betalains in edible fruits of three cactaceae taxa—*Epiphyllum, Hylocereus*, and *Opuntia*—Their LC-MS/MS and FTIR identification and biological activities. Plants.

[41] Mata A., Ferreira J.P., Semedo C., Serra T., Duarte C.M.M., Bronze M.R. (2016). Contribution to the characterization of *Opuntia* spp. juices by LC–DAD–ESI-MS/MS. Food Chem..

[42] Zhang H., Chen G., Yang J., Yang C., Guo M. (2022). Screening and characterisation of potential antioxidant, hypoglycemic and hypolipidemic components revealed in *Portulaca oleracea via* multi-target affinity. Phytochem. Anal..

[43] Farag M.A., Shakour Z.T.A. (2019). Metabolomics driven analysis of 11 *Portulaca* leaf taxa as analysed *via* UPLC-ESI-MS/MS and chemometrics. Phytochemistry.

[44] Gök H.N., Luca S.V., Ay S.T., Komsta Ł., Salmas R.E., Orhan I.E., Skalicka-Woźniak K. (2022). Profiling the annual change of the neurobiological and antioxidant effects of five *Origanum* species in correlation with their phytochemical composition. Food Chem..

[45] Taamalli A., Arráez-Román D., Abaza L., Iswaldi I., Fernández-Gutiérrez A., Zarrouk M., Segura-Carretero A. (2015). LC-MS-based metabolite profiling of methanolic extracts from the medicinal and aromatic species *Mentha pulegium* and *Origanum majorana*. Phytochem. Anal..

[46] Hossain M.B., Rai D.K., Brunton N.P., Martin-Diana A.B., Barry-Ryan C. (2010). Characterization of phenolic composition in *Lamiaceae* spices by LC-ESI-MS/MS. J. Agric. Food Chem..

[47] Razgonova M., Zakharenko A., Pikula K., Manakov Y., Ercisli S., Derbush I., Golokhvast K. (2021). LC-MS/MS screening of phenolic compounds in *Wild* and cultivated grapes *Vitis amurensis* Rupr. Molecules.

[48] Das Neves Costa F., Jerz G., Hewitson P., De Souza Figueiredo F., Ignatova S. (2021). Laguncularia racemosa phenolics profiling by three-phase solvent system step-gradient using high-performance countercurrent chromatography with off-line. Molecules.

[49] Das Neves Costa F., Borges R.M., Leitão G.G., Jerz G. (2019). Preparative mass-spectrometry profiling of minor concentrated metabolites in *Salicornia gaudichaudiana* Moq by high-speed countercurrent chromatography. J. Sep. Sci..

[50] Ziani B.E.C., Barros L., Boumehira A.Z., Bachari K., Heleno S.A., Alves M.J., Ferreira I.C. (2018). Profiling polyphenol composition by HPLC-DAD-ESI/MSn and the antibacterial activity of infusion preparations obtained from four medicinal plants. Food Funct..

[51] De Freitas Laiber Pascoalag G., de Almeida Sousa Cruza M.A., de Abreu J.P., Santos M.C.B., Fanaro G.B., Maróstica M.R., Silva O.F., Moreira R.F.A., Cameron L.C., Ferreira M.S.L. (2022). Evaluation of the antioxidant capacity, volatile composition and phenolic content of hybrid *Vitis vinifera* L. varieties sweet sapphire and sweet surprise. J. Food Chem..

[52] Marchi R.C., Campos I.A.S., Santana V.T., Carlos R.M. (2022). Chemical implications and considerations on techniques used to assess the in vitro antioxidant activity of coordination compounds. Coord. Chem. Rev..

[53] Da Silva D.V.T., dos Santos Baião D., de Oliveira Silva F., Alves G., Perrone D., Del Aguila E.M., Paschoalin V.M.F. (2019). Betanin, a natural food additive: Stability, bioavailability, antioxidant and preservative ability assessments. Molecules.

[54] Esatbeyoglu T., Wagner A.E., Schini-Kerth V.B., Rimbach G. (2015). Betanin—A food colorant with biological activity. Mol. Nutr. Food Res..

[55] Zhang L., Zhang P., Xia C., Cheng Y., Guo X., Li Y. (2020). Effects of malic acid and citric acid on growth performance, antioxidant capacity, haematology and immune response of *Carassius auratus* gibelio. Aquac. Res..

[56] Fiume Z. (2001). Final report on the safety assessment of malic acid and sodium malate. Int. J. Toxicol..

[57] Aluko R.E. (2015). Amino acids, peptides, and proteins as antioxidants for food preservation. Preservation.

[58] Velika B., Kron I. (2012). Antioxidant properties of benzoic acid derivatives against superoxide radical. Free Radic..

[59] Teixeira J., Gaspar A., Garrido E.M., Garrido J., Borges F. (2013). Hydroxycinnamic acid antioxidants: An electrochemical overview. Biomed Res. Int..

[60] Adomako-Bonsu A.G., Chan S.L., Pratten M., Fry J.R. (2017). Antioxidant activity of rosmarinic acid and its principal metabolites in chemical and cellular systems: Importance of physico-chemical characteristics. Toxicol. In Vitro.

[61] Sahakyan N., Bartoszek A., Jacob C., Petrosyan M., Trchounian A. (2020). Bioavailability of tannins and other oligomeric polyphenols: A still to be studied phenomenon. Curr. Pharmacol. Rep..

[62] Maki K.C., Orloff D.G., Nicholls S.J., Dunbar R.L., Roth E.M., Curcio D., Johnson J., Kling D., Davidson M.H. (2013). A highly bioavailable omega-3 free fatty acid formulation improves the cardiovascular risk profile in high-risk, statin-treated patients with residual hypertriglyceridemia (the ESPRIT trial). Clin. Ther..

[63] Mohadjerani M., Tavakoli R., Hosseinzadeh R. (2014). Fatty acid composition, antioxidant and antibacterial activities of *Adonis wolgensis* L. extract. Avicenna J. Phytomed..

[64] Panche A.N., Diwan A.D., Chandra S.R. (2016). Flavonoids: An overview. J. Nutr. Sci..

[65] Thilakarathna S.H., Rupasinghe H.V. (2013). Flavonoid bioavailability and attempts for bioavailability enhancement. Nutrients.

[66] Pankey G.A., Sabath L.D. (2004). Clinical relevance of bacteriostatic versus bactericidal mechanisms of action in the treatment of Gram-positive bacterial infections. Clin. Infect. Dis..

[67] Ercisli S., Coruh I., Gormez A., Sengul M. (2008). ntioxidant and antibacterial activities of *Portulaca oleracea* L. grown wild in Turkey. Ital. J. Food Sci..

[68] Parthasarathy A., Borrego E.J., Savka M.A., Dobson R.C., Hudson A.O. (2021). Amino acid–derived defense metabolites from plants: A potential source to facilitate novel antimicrobial development. J. Biol. Chem..

[69] Herald P.J., Davidson P.M. (1983). Antibacterial activity of selected hydroxycinnamic acids an effect of positional isomerism of benzoic acid derivatives on antibacterial activity against *Escherichia coli*. J. Food Sci..

[70] Shamsudin N.F., Ahmed Q.U., Mahmood S., Ali Shah S.A., Khatib A., Mukhtar S., Alsharif M.A., Parveen H., Zakaria Z.A. (2022). Antibacterial effects of flavonoids and their structure-activity relationship study: A comparative interpretation. Molecules.

[71] Geran R.I., Greenberg N.H., Macdonald M.M., Schumacher A.M., Abbott B.J. (1972). Protocols for screening chemical agents and natural products against animal tumors and other biological systems. Cancer Chemother. Rep..

[72] Zengin G., Mahomoodally M.F., Rocchetti G., Lucini L., Sieniawska E., Świątek Ł., Rajtar B., Polz-Dacewicz M., Senkardes I., Aktümsek A. (2020). Chemical characterization and bioactive properties of different extracts from *Fibigia clypeata*, an unexplored plant food. Foods.

